# Supplementary material for: The maluma/takete effect is late: No longitudinal evidence for shape sound symbolism in the first year
Source: PLoS One. 2023 Nov 9;18(11):e0287831. doi: 10.1371/journal.pone.0287831 (PMC10635456; doi:10.1371/journal.pone.0287831)
Supplement: S1 File — (PDF) [file pone.0287831.s001.pdf]

Supplementary Analyses

Table S1

Logistic Mixed Effects Regression Model Predicting Congruent Shape Choices with Nonword Type, in Experiment 1a

Fixed Effect	B	SE	t	p
Intercept	0.47	0.25	1.86	.06
Nonword Type	1.23	0.32	3.79	<.001***
Random Effect			s^2	
Participant Intercept			0.068	

Table S2

Logistic Mixed Effects Regression Model Predicting Congruent Shape Choices with Nonword Type, in Experiment 1b

Fixed Effect	B	SE	t	p
Intercept	0.97	0.50	1.94	.053
Nonword Type	2.12	1.05	2.01	.04*
Random Effect		s^2		r
Participant Intercept		1.18		
Participant Nonword Type Slope		2.10		.14
Item Intercept		0.29		

Table S3

Linear Mixed Effects Regression Model Predicting Looking Time for All Infants With Nonword Type, Categorical Visit and Phoneme Type, in Experiment 2

Fixed Effect	B	SE	t	p
Intercept	-0.07	0.01	-12.35	<.001***
Nonword Type	0.01	0.01	0.79	.43
Visit (2 vs 1)	0.03	0.01	2.16	.03*
Visit (3 vs 2)	0.01	0.01	0.37	.71
Phoneme Type	0.00	0.01	-0.22	.83
Nonword Type x Visit (2 vs 1)	0.00	0.03	0.18	.86
Nonword Type x Visit (3 vs 2)	0.03	0.03	0.96	.34
Nonword Type x Phoneme Type	0.01	0.02	0.67	.50
Visit (2 vs 1) x Phoneme Type	0.00	0.03	0.14	.89
Visit (3 vs 2) x Phoneme Type	0.00	0.03	0.05	.96
Nonword Type x Visit (2 vs 1) x Phoneme Type	-0.04	0.05	-0.85	.39
Nonword Type x Visit (3 vs 2) x Phoneme Type	0.00	0.06	-0.08	.94
Random Effect		s^2		
Participant Intercept		0.0003		

Table S4

Linear Mixed Effects Regression Model Predicting Looking Time for All Infants With Nonword Type, Continuous Ages and Phoneme Type, in Experiment 2

Fixed Effect	B	SE	t	p
(Intercept)	-0.07	0.01	-12.50	<.001***
Nonword Type	0.01	0.01	0.68	.50
Age	0.01	0.01	2.41	.02*
Phoneme Type	0.00	0.01	-0.23	.82
Nonword Type x Age	0.01	0.01	1.04	.30
Nonword Type x Phoneme Type	0.02	0.02	0.75	.46
Age x Phoneme Type	0.00	0.01	-0.15	.89
Nonword Type x Age x Phoneme Type	-0.02	0.02	-0.94	.35
Random Effect		s^2		
Participant Intercept		0.0003		

Table S5

Linear Mixed Effects Regression Model Predicting Looking Time for All Infants With Nonword Type and Categorical Visit Including Infants Who Attended All Sessions, in Experiment 2

Fixed Effect	B	SE	t	p
Intercept	-0.07	0.01	-12.45	<.001***
Nonword Type	0.01	0.01	0.60	.55
Visit (2 vs 1)	0.03	0.01	2.07	.04*
Visit (3 vs 2)	0.01	0.01	0.40	.69
Nonword Type x Visit (2 vs 1)	0.02	0.03	0.58	.56
Nonword Type x Visit (3 vs 2)	0.02	0.03	0.86	.39
Random Effect			s^2	

Participant Intercept	0.0001

Table S6

Linear Mixed Effects Regression Model Predicting Looking Time for all Infants With Nonword Type and Age as a Continuous Variable Including Infants Who Attended All Sessions, in Experiment 2

Fixed Effect	b	SE	t	p
(Intercept)	-0.07	0.01	12.36	<.001***
Nonword Type	0.01	0.01	0.60	.55
Age	0.01	0.01	2.31	.02*
Nonword Type x Age	0.02	0.01	1.40	.16
Random Effect	s^2			
Participant Intercept	0.0003			

Table S7

Linear Mixed Effects Regression Model Predicting Looking Time for All Infants With Nonword Type and Categorical Visit, Excluding Cheechee Trials, in Experiment 2

Fixed Effect	B	SE	t	p
Intercept	-0.07	0.01	-10.65	<.001***
Nonword Type	0.00	0.01	0.27	.78
Visit (2 vs 1)	0.02	0.02	1.40	.16
Visit (3 vs 2)	0.00	0.02	0.24	.82
Nonword Type x Visit (2 vs 1)	0.02	0.03	0.58	.57

Nonword Type x Visit (3 vs 2)	0.03	0.03	0.85	.40
Random Effect			s^2	
Participant Intercept			0.00	

Table S8

Linear Mixed Effects Regression Model Predicting Looking Time for All Infants With Nonword Type and Continuous Age, Excluding Cheechee Trials, in Experiment 2

Fixed Effect	B	SE	t	p
(Intercept)	0.43	0.01	64.96	<.001***
Nonword Type	0.00	0.01	0.15	.88
Age	0.01	0.01	1.67	.10
Nonword Type x Age	0.02	0.01	1.21	.23
Random Effect			s^2	
Participant Intercept			0.00	

Table S9

Factor loadings of babbling scores.

Phoneme	Factor Loading	Communality	Uniqueness
/tʃ/	0.97	0.93	0.068
/k/	0.93	0.87	0.126
/l/	0.77	0.59	0.406

/oʊ/	0.68	0.46	0.539
/eɪ/	0.64	0.41	0.589

Table S10

Factor loadings of physical milestones.

Milestone	Factor Loading	Communality	Uniqueness
Going Up and Down Stairs	0.97	0.93	0.068
Walking with Support	0.93	0.87	0.126
Walking Alone Easily	0.77	0.59	0.406
