# Supplementary material for: The maluma/takete effect is late: No longitudinal evidence for shape sound symbolism in the first year
Source: PLoS One. 2023 Nov 9;18(11):e0287831. doi: 10.1371/journal.pone.0287831 (PMC10635456; doi:10.1371/journal.pone.0287831)
Supplement: Attachment — Submitted filename: PONE-D-22-33843_Comments.pdf [file pone.0287831.s003.pdf]

PLOS ONE

The Maluma/Takete Effect is Late No Longitudinal Evidence for Shape Sound Symbolism in the First Year

--Manuscript Draft--

**Manuscript Number:**	PONE-D-22-33843
**Article Type:**	Research Article
**Full Title:**	The Maluma/Takete Effect is Late No Longitudinal Evidence for Shape Sound Symbolism in the First Year
**Short Title:**	Shape Sound Symbolism in the First Year
**Corresponding Author:**	David Sidhu<br>Carleton University<br>Ottawa, Ontario CANADA
**Keywords:**	sound symbolism; iconicity; cognitive psychology; psycholinguistics; developmental psychology; language; bouba/kiki effect; maluma/takete effect; psychology of language; semantics; phonology; crossmodal correspondence
**Abstract:**	The maluma/takete effect refers to an association between certain language sounds (e.g., /m/ and /o/) and round shapes, and other language sounds (e.g., /t/ and /i/) and spiky shapes. This is an example of sound symbolism and stands in opposition to arbitrariness of language. It is still unknown when sensitivity to sound symbolism arises. In the present series of studies, we first confirmed that the classic maluma/takete effect would be observed in adults using our novel 3-D object stimuli (Experiments 1a and 1b). We then conducted the first longitudinal test of the maluma/takete effect, testing infants at 4-, 8- and 12-months of age (Experiment 2). Sensitivity to sound symbolism was measured with a looking time preference task, in which infants were shown images of a round and a spiky 3-D object while hearing either a round- or spiky-sounding nonword. We did not detect a significant difference in looking time based on nonword type. We also collected a series of individual difference measures including measures of vocabulary, movement ability and babbling. Analyses of these measures revealed that 12-month olds who babbled more showed a greater sensitivity to sound symbolism. Finally, in Experiment 3, we had parents take home round or spiky 3-D printed objects, to present to 7- to 8-month-old infants paired with either congruent or incongruent nonwords. This language experience had no effect on subsequent measures of sound symbolism sensitivity. Taken together these studies demonstrate that sound symbolism is elusive in the first year, and shed light on the mechanisms that may contribute to its eventual emergence.
**Order of Authors:**	David Sidhu
	Angeliki Athanasopoulou
	Stephanie L. Archer
	Natalia Czarnecki
	Suzanne Curtin
	Penny M. Pexman
**Additional Information:**	
**Question**	**Response**
**Financial Disclosure**<br><br>Enter a financial disclosure statement that describes the sources of funding for the work included in this submission. Review the submission guidelines for detailed requirements. View published research	This research was supported by the Social Sciences and Humanities Research Council of Canada (SSHRC; https://www.sshrc-crsh.gc.c) through an Insight Development Grant (430-2017-00003) to PP and SC. The funders had no role in study design, data collection and analysis, decision to publish, or preparation of the manuscript.

Powered by Editorial Manager® and ProduXion Manager® from Aries Systems Corporation

articles from *PLOS ONE* for specific examples.<br><br>This statement is required for submission and **will appear in the published article** if the submission is accepted. Please make sure it is accurate.<br><br>**Unfunded studies**<br>Enter: *The author(s) received no specific funding for this work.*<br><br>**Funded studies**<br>Enter a statement with the following details:<br>• Initials of the authors who received each award<br>• Grant numbers awarded to each author<br>• The full name of each funder<br>• URL of each funder website<br>• Did the sponsors or funders play any role in the study design, data collection and analysis, decision to publish, or preparation of the manuscript?<br>• **NO** - Include this sentence at the end of your statement: *The funders had no role in study design, data collection and analysis, decision to publish, or preparation of the manuscript.*<br>• **YES** - Specify the role(s) played.<br><br>* typeset	
**Competing Interests**<br><br>Use the instructions below to enter a competing interest statement for this submission. On behalf of all authors, disclose any competing interests that could be perceived to bias this work—acknowledging all financial support and any other relevant financial or non-financial competing interests.<br><br>This statement is required for submission and **will appear in the published article** if the submission is accepted. Please make sure it is accurate and that any funding sources listed in your Funding Information later in the submission form are also declared in your Financial Disclosure statement.	The authors have declared that no competing interests exist.

View published research articles from *PLOS ONE* for specific examples.<br><br>**NO authors have competing interests**<br><br>Enter: *The authors have declared that no competing interests exist.*<br><br>**Authors with competing interests**<br><br>Enter competing interest details beginning with this statement:<br><br>*I have read the journal's policy and the authors of this manuscript have the following competing interests: [insert competing interests here]*<br><br>* typeset	
**Ethics Statement**<br><br>Enter an ethics statement for this submission. This statement is required if the study involved:<br><br>• Human participants<br>• Human specimens or tissue<br>• Vertebrate animals or cephalopods<br>• Vertebrate embryos or tissues<br>• Field research<br><br>Write "N/A" if the submission does not require an ethics statement.<br><br>General guidance is provided below. Consult the submission guidelines for detailed instructions. **Make sure that all information entered here is included in the Methods section of the manuscript.**	All studies were approved by the Research Ethics Board at the University of Calgary. Written (Experiment 1a) or electronic consent (Experiment 1b) was received from adult participants. Written consent (Experiments 2 and 3) was received from infants' caregivers.

**Format for specific study types**<br><br>**Human Subject Research (involving human participants and/or tissue)**<br>• Give the name of the institutional review board or ethics committee that approved the study<br>• Include the approval number and/or a statement indicating approval of this research<br>• Indicate the form of consent obtained (written/oral) or the reason that consent was not obtained (e.g. the data were analyzed anonymously)<br><br>**Animal Research (involving vertebrate animals, embryos or tissues)**<br>• Provide the name of the Institutional Animal Care and Use Committee (IACUC) or other relevant ethics board that reviewed the study protocol, and indicate whether they approved this research or granted a formal waiver of ethical approval<br>• Include an approval number if one was obtained<br>• If the study involved *non-human primates*, add *additional details* about animal welfare and steps taken to ameliorate suffering<br>• If anesthesia, euthanasia, or any kind of animal sacrifice is part of the study, include briefly which substances and/or methods were applied<br><br>**Field Research**<br><br>Include the following details if this study involves the collection of plant, animal, or other materials from a natural setting:<br>• Field permit number<br>• Name of the institution or relevant body that granted permission	
**Data Availability**<br><br>Authors are required to make all data underlying the findings described fully available, without restriction, and from the time of publication. PLOS allows rare exceptions to address legal and ethical concerns. See the PLOS Data Policy and FAQ for detailed information.	Yes - all data are fully available without restriction

A Data Availability Statement describing where the data can be found is required at submission. Your answers to this question constitute the Data Availability Statement and **will be published in the article**, if accepted.<br><br>**Important:** Stating 'data available on request from the author' is not sufficient. If your data are only available upon request, select 'No' for the first question and explain your exceptional situation in the text box.<br><br>Do the authors confirm that all data underlying the findings described in their manuscript are fully available without restriction?	
**Describe where the data may be found in full sentences. If you are copying our sample text, replace any instances of XXX with the appropriate details.**<br><br>• If the data are **held or will be held in a public repository**, include URLs, accession numbers or DOIs. If this information will only be available after acceptance, indicate this by ticking the box below. For example: *All XXX files are available from the XXX database (accession number(s) XXX, XXX.*).<br>• If the data are all contained **within the manuscript and/or Supporting Information files**, enter the following: *All relevant data are within the manuscript and its Supporting Information files.*<br>• If neither of these applies but you are able to provide **details of access elsewhere**, with or without limitations, please do so. For example:<br><br>*Data cannot be shared publicly because of [XXX]. Data are available from the XXX Institutional Data Access / Ethics Committee (contact via XXX) for researchers who meet the criteria for access to confidential data.*<br><br>*The data underlying the results presented in the study are available from (include the name of the third party*	All data and analysis files are available from the OSF database (https://osf.io/sd62f/).

and contact information or URL).<br>• This text is appropriate if the data are owned by a third party and authors do not have permission to share the data.<br><br>* typeset	
Additional data availability information:	

Powered by Editorial Manager® and ProduXion Manager® from Aries Systems Corporation

Click here to access/download;Manuscript;CCLL Manuscript v11.docx

The Maluma/Takete Effect is Late:

No Longitudinal Evidence for Shape Sound Symbolism in the First Year

David M. Sidhu[1], Angeliki Athanasopoulou[2], Stephanie L. Archer[3], Natalia Czarnecki[4], Suzanne Curtin[5], & Penny M. Pexman[4]

[1] Department of Psychology, Carleton University

[2] School of Languages, Linguistics, Literatures, and Cultures, University of Calgary

[3] Department of Linguistics, University of Alberta

[4] Department of Psychology, University of Calgary

[5] Department of Child and Youth Studies, Brock University

Abstract

The maluma/takete effect refers to an association between certain language sounds (e.g., /m/ and /o/) and round shapes, and other language sounds (e.g., /t/ and /i/) and spiky shapes. This is an example of *sound symbolism* and stands in opposition to arbitrariness of language. It is still unknown when sensitivity to sound symbolism arises. In the present series of studies, we first confirmed that the classic maluma/takete effect would be observed in adults using our novel 3-D object stimuli (Experiments 1a and 1b). We then conducted the first longitudinal test of the maluma/takete effect, testing infants at 4-, 8- and 12-months of age (Experiment 2). Sensitivity to sound symbolism was measured with a looking time preference task, in which infants were shown images of a round and a spiky 3-D object while hearing either a round- or spiky-sounding nonword. We did not detect a significant difference in looking time based on nonword type. We also collected a series of individual difference measures including measures of vocabulary, movement ability and babbling. Analyses of these measures revealed that 12-month olds who babbled more showed a greater sensitivity to sound symbolism. Finally, in Experiment 3, we had parents take home round or spiky 3-D printed objects, to present to 7- to 8-month-old infants paired with either congruent or incongruent nonwords. This language experience had no effect on subsequent measures of sound symbolism sensitivity. Taken together these studies demonstrate that sound symbolism is elusive in the first year, and shed light on the mechanisms that may contribute to its eventual emergence.

Introduction

For the most part, the sound of a word is arbitrarily associated with its meaning. As an example, one must learn that the set of speech sounds in *cat* refers to a feline with whiskers and claws. The speech sounds themselves do nothing to help in this process. This principle of arbitrariness was once thought to be more or less ubiquitous in language (e.g., [1]). However, there is growing appreciation for the role of *iconicity* in language: instances in which speech sounds imitate or depict meanings (see [2, 3]).[1] For instance, the speech sounds in *meow* do more than serve as a set of sounds to associate with a meaning (as with *cat*). They also imitate the sound to which they refer, making *meow* an iconic word.

Importantly, iconicity can emerge beyond the imitation of sounds. Speech sounds can also imitate non-sound features through analogy. For example, the speech sounds in *cactus* are abrupt, with sudden changes in amplitude [4]. In this way, *cactus* imitates the visual property of spikiness through analogy. The term for this is *sound symbolism*: associations between speech sounds and perceptual and/or semantic properties. In this paper we focus on the most well-studied instance of sound symbolism: associations between speech sounds and either round or spiky shapes (the *maluma/takete effect*; [5]). For instance, when adults are shown shapes like those in Figure 1 and asked which is a *maluma* and which is a *takete*, roughly 90% of individuals will respond that the round shape is *maluma* while the spiky shape is *takete* [6]. In general, sonorants (e.g., /l/), voiced stops (e.g., /b/), unvoiced fricatives (e.g., /s/), and rounded-back vowels (e.g., /u/) tend to be associated with round shapes [7,8]. Conversely, voiceless stops (e.g., /k/), affricates (e.g., /tʃ/) and front-unrounded vowels (e.g., /i/) tend to be associated with spiky

[1] While we focus here on the sounds of spoken language, iconicity is much more far reaching. It can emerge in tone of voice or gesture. It also plays an important role in sign language.

shapes [7]. These associations emerge on forced choice tasks in a bias to pair nonwords with certain shapes (e.g., [9]), on implicit tasks in facilitated responses to congruent nonword-shape pairings (e.g., [10]), and in different neural responses to congruent vs. incongruent pairings (e.g., [11]). In this study we address a pair of unanswered questions. When do children become sensitive to shape sound symbolism? And what developmental milestones are related to that sensitivity?

Missing point: Lit review suggests this effect among 90% of participants in all language contexts even [illegible]h one of the cited papers shows that the 'canonical' word forms do not work in all language [illegible]xts. Other work shows that the effect can take a different form in different languages, so [illegible]earning may be part of the trajectory for this effect.

Figure [illegible]

Examples of Shapes Used in Studies of the Maluma/Takete Effect

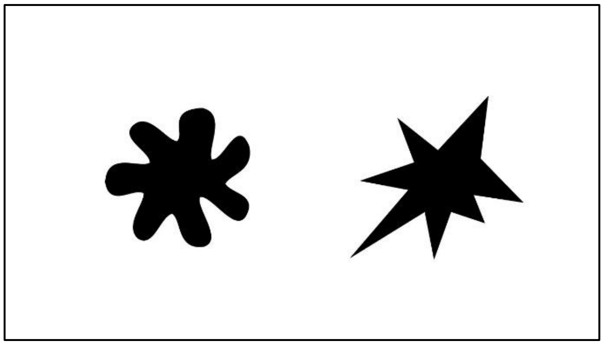

These questions are theoretically important for several reasons. For one, some have proposed that sound symbolism and iconicity play a role in language acquisition (e.g., [12]; for a review see [13]). There is now a wealth of evidence that iconic words are easier for children to acquire ([14, 15]; termed *local enhancement* by [13]). It could be that the imitative links in iconic words help children associate a sound with a meaning. Imai et al. [16] also found evidence that infants had an easier time generalizing an iconic label to a novel instance of the word's referent (e.g., learning that a certain kind of movement is called *batobato* whether it is performed by a

rabbit or a bear). Some have also made the broader claim that iconicity can provide general benefits to word learning. For example, Imai and Kita [12] speculated that the existence of iconic words could help children realize that sounds refer to things in the world (i.e., gain referential insight). However, there has not been evidence for this *general enhancement* (reviewed in [13]). Knowing *when* infants become sensitive to sound symbolism can help understand its contribution to word learning. For example, it would be informative to know whether sensitivity develops before or after referential insight.

Knowing when sensitivity to sound symbolism develops with regards to developmental milestones and experiences would also be informative for theories of sound symbolism. It is still unclear which linguistic aspects play a primary role in the maluma/takete effect. It could be that the phonetic properties of speech sounds play a key role (e.g., that /t/ is associated with spiky things because of the sudden changes in amplitude). One could also make a case for experiences of articulation (e.g., that /t/ is associated with spiky things because of the abrupt release of the articulator after the closure). One might also suggest that the shapes of letters (e.g., the round "o") or the lips (e.g., the lips for rounded vowels) play important roles in the effect (for discussions of these different possibilities see [17-21]). Knowing if sensitivity to sound symbolism emerges before or after experience with these different linguistic aspects could thus be informative.

I think Ramachandran and Hubbard were the first to make this proposal about articulatory congruence particularly for lip shape but not included in this ref list

In addition, it is not yet known *how* speech sounds get associated with shapes. For instance, some have suggested that sound symbolic associations arise from an internalization of statistical occurrences in the environment [4,22]. Others have proposed that the maluma/takete effect could stem from knowledge of patterns in language, in which certain sounds occur in words for certain shapes [23,24]. Testing whether sensitivity to sound symbolism emerges before

or after infants begin exploring the environment or acquiring vocabulary would shed light on these possibilities.

Previous Work on Infant Sound Symbolism

There have been previous attempts to investigate whether infants of different ages are sensitive to sound symbolism. Maurer et al. [25] gave two and a half year olds the sort of task illustrated in Figure 1, and found that infants' responses were comparable with those of adults, thus suggesting that sensitivity emerges by 2.5 years of age. In order to test younger preverbal infants, researchers have instead used techniques that do not require participants to understand instructions or make verbal responses. The most common approach has been a looking time preference task. Infants sit on a caregiver's lap and have their attention directed towards a screen showing either one shape or two (one round and one spiky). While looking at the screen, infants hear a round- or a spiky-associated nonword. In the one-shape version, the dependent measure is the amount of time infants look at the screen for a congruent vs. incongruent nonword. In the two-shape version, the dependent measure is looking time to the congruent vs. incongruent shape.

Also relevant: This procedure was non-blind to experimenter so children might be picking up other subtle paralinguistic or prosodic cues when identifying the expected response.

Ozturk et al. [26] used a one-shape looking time preference task with four-month-olds. They found that infants looked longer at the incongruent pairings as compared to the congruent pairings, suggesting very early sensitivity to sound symbolism. However, this experiment only included 12 infants. In addition, two follow up experiments that presented nonwords contrasting only in either vowels or consonants did not find a significant effect. Pejovic and Molnar [27] attempted to replicate this effect with a sample of 26 four-month-olds using the same task, and found no differences between congruent and incongruent pairings. However, they did find a significant difference in a sample of 12-month-olds (n = 26).

There is also neuroimaging evidence of sensitivity to sound symbolism emerging by 12 months. Asano et al. [11] studied 49 11-month-olds with electroencephalogram (*EEG*) based measures of brain activity. Participants were first shown a shape and then heard a congruent or incongruent nonword. The researchers found differences between congruent and incongruent pairings in phase synchronization, and in the intensity of infants' N400 responses (a negative going waveform sensitive to semantic integration). This finding was complimented by that of a study of 22 11-month-olds using near infrared spectroscopy (NIRS) to measure brain activity [28]. This study found greater activity in the right superior temporal sulcus for congruent pairings of *round* nonwords. Importantly, this area has been implicated in the processing of iconicity in adults [29,30].

The literature on infant sound symbolism was recently summarized in a meta-analysis by Fort et al. [31]. They combined a total of 44 effect sizes, from 22 different studies, which included infants ranging in age from 4-38 months. The studies included both behavioural (e.g., forced choice and looking time) and neural measures. Fort et al. found that the effect of sound symbolism (i.e., comparing responses to congruent vs. incongruent pairings) was significantly different than zero. However, after adding nonword type (i.e., round or spiky), age, their interaction, and publication status, the effect of sound symbolism was no longer significant. They did, however, find a larger effect for round vs. spiky nonwords. When analyzing each nonword type separately, they found a marginal effect of sound symbolism for round nonwords (p = .062), but not spiky nonwords. Notably, age was a significant predictor in the case of spiky nonwords, suggesting that sensitivity to spiky sound symbolism increases with age.

In sum, the literature on infant sound symbolism is equivocal. Many studies have included small samples: the 22 published studies examined by Fort et al. [31] included an

average of just over 19 participants. Overall, after including control variables, there was not a significant effect of sound symbolism in Fort et al.'s meta-analysis. However, some of the studies reviewed above suggest that there is an emerging sensitivity to sound symbolism by about 12 months of age. In the present study we conducted a thorough test of infants' emerging sensitivity to sound symbolism, using a longitudinal design to investigate whether that sensitivity emerges in the first year of life and what milestone or experience might be associated with its emergence.

The Present Study

In the present study we first verified that the maluma/takete effect will be observed in adults using our stimuli (Experiments 1a and 1b). Then we conducted a longitudinal experiment testing sound symbolism at 4, 8 and 12 months (Experiment 2). We measured several individual differences at each time point to gain insight into the developmental milestones and experiences that are related to sound symbolism. We collected measures of infants' babbling to quantify their experience articulating speech sounds. We also included a measure of their ability to move through the environment, to quantify their tactile experience of the world, and their knowledge of the statistical regularities in the world. Finally, we measured infants' receptive and productive vocabulary, to quantify their overall language development, and to estimate their knowledge of patterns in language. Finally, in Experiment 3, we conducted a study in which we directly manipulated the statistical regularities in the environment. Caregivers either produced congruent or incongruent labels for a round or a spiky shape at home. We then examined the effect of this on infants' sound symbolic associations.

Experiment 1a

Method

Participants

Participants were 30 undergraduates at the University of Calgary who participated in exchange for course credit.

Not collected at the time of test? or not retained?

Records of demographics for these participants were not kept. All participants reported English fluency and normal or corrected to normal vision.

Stimuli

We used the same shape stimuli in each of the experiments reported here. Because these needed to be taken home by families in Experiment 3, we created 3D-printed models of a round and a spiky shape. These were painted either green or orange. The visual stimuli in Experiments 1 and 2 were photographs of these shapes, see Figure 2. On each trial in Experiment 1a participants saw a round and a spiky shape of the same colour. There were two different versions of each same colour pair, with the objects photographed from different angles. This created eight shape pairs. We collected pilot ratings on these images from a separate group of adult participants (n = 12) to ensure that the shapes paired in each round/spiky image pair were similar in rated visual interest.

Figure 2

Examples of Round and Spiky Object Stimuli

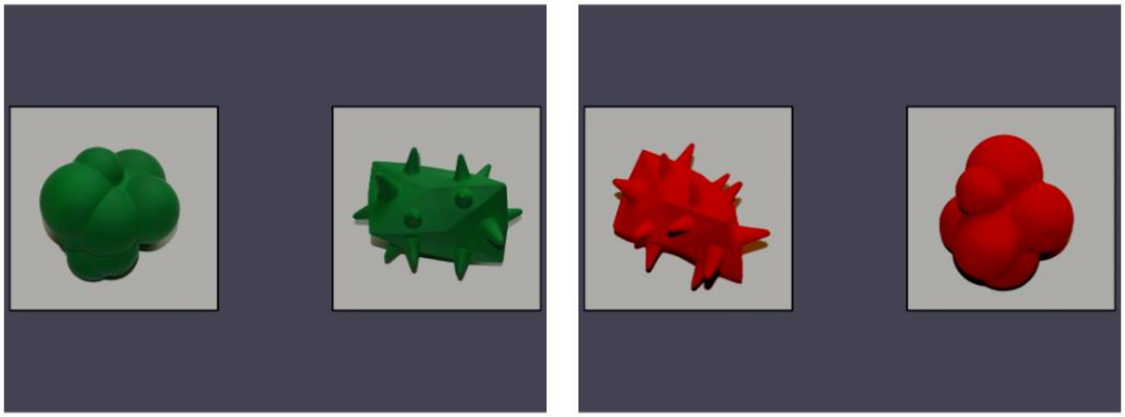

As in previous studies of infant sound symbolism (e.g., [26,27]), nonword auditory stimuli consisted of four disyllabic CVCV nonwords. Two of these (i.e., /bobo/ and /lulu/) contained round-associated phonemes (voiced stops or sonorant consonants; and rounded back vowels), and two (i.e., /keke/ and /tʃitʃi/) contained spiky-associated phonemes (voiceless stops or affricate consonants; and unrounded front vowels; [7]). In addition, for the purpose of our subsequent experiments with children, one of each type of nonword contained consonants that are relatively more early-acquired, in that children typically produce them around three years of age (i.e., /b/ and /k/), and one of each contained consonants that are relatively later-acquired, in that children typically produce them around 4.5 years of age (i.e., /l/ and /tʃ/; [32]). Nonwords were recorded by a Canadian-English female native speaker in infant directed speech.

Procedure

Participants took part in person in our laboratory at the University of Calgary. The experiment was run using E-Prime 2.0 software. Participants wore sound attenuating headphones. On each trial, participants heard a nonword and this was repeated until an eventual response was made (to match the stimulus repetition in the planned infant experiment). Participants were also presented with a shape pair as the audio began, and were asked to decide which shape best fit the nonword. Responses were made via button box. Stimuli order, and the pairing between nonwords and shape pairs, was randomized across participants.

Is presentation script shared?

Are audio available?

Are image stimuli shared?

Results

All mixed effect models were computed using the “lme4” [33] and “afex” [34] packages in the statistical software R [35]. Here and in subsequent experiments, we included random intercepts for subject and item. We also included a random subject slope for predictor(s) of interest. To deal with convergence issues and singular fits we attempted these remedies, in the following order: increasing the number of iterations, switching to the “bobyqa” optimizer, removing the correlation between the random subject intercept and slope, removing the random subject slope, and removing the random intercept with the lowest associated variance (see [36,37]). Data and analysis code for all expeirments can be found here: https://osf.io/sd62f/.

The present analysis began as a logistic mixed effects regression. The dependent variable was whether or not a participant chose the round shape on a given trial. The predictor was nonword type (using dummy coding; spiky [0] vs. round [+1]). However, there was no variance in the random effects, and so we proceeded with a simple logistic regression. The analysis revealed a significant effect of nonword type such that participants were 6.68 times more likely to choose the round shape for a round vs. a spiky nonword (b = 1.90, p < .001). See Figure 3.

Is the 'simple' regression still a repeated measures design? This is important because one person's responses may be correlated more closely to their own responses than to others' responses - even without the random effect being significant (i.e., people respond differently to one another). By keeping the random factor in your model, you may actually improve the power to detect an effect. Perhaps there is different reasoning behind this decision though, so I'd be happy to understand more about it - may be in a footnote or supplementary materials. I couldn't see where to go for additional a full statistical reports of the outcomes of the models in the OSF archive, so it would be great to make that clearly linked in this section.

Please add a measure of confidence around this point estimate (e.g., CI?) or clarify

where can we find details of the analysis and how it was implemented?

Participants chose the sound symbolically congruent shape on 71% of trials. A follow up analysis suggested that congruent choices were made significantly more often on round (82%) vs spiky (60%) trials (b = 1.09, p < .001). See Table 1 for results by item.

This graph would be better if it were possible to view individual data points or distributions in the format of density plots

Figure 3

Proportion of Trials on Which Adult Participants Chose the Round Shape of the Pair in Experiments 1a and 1b.

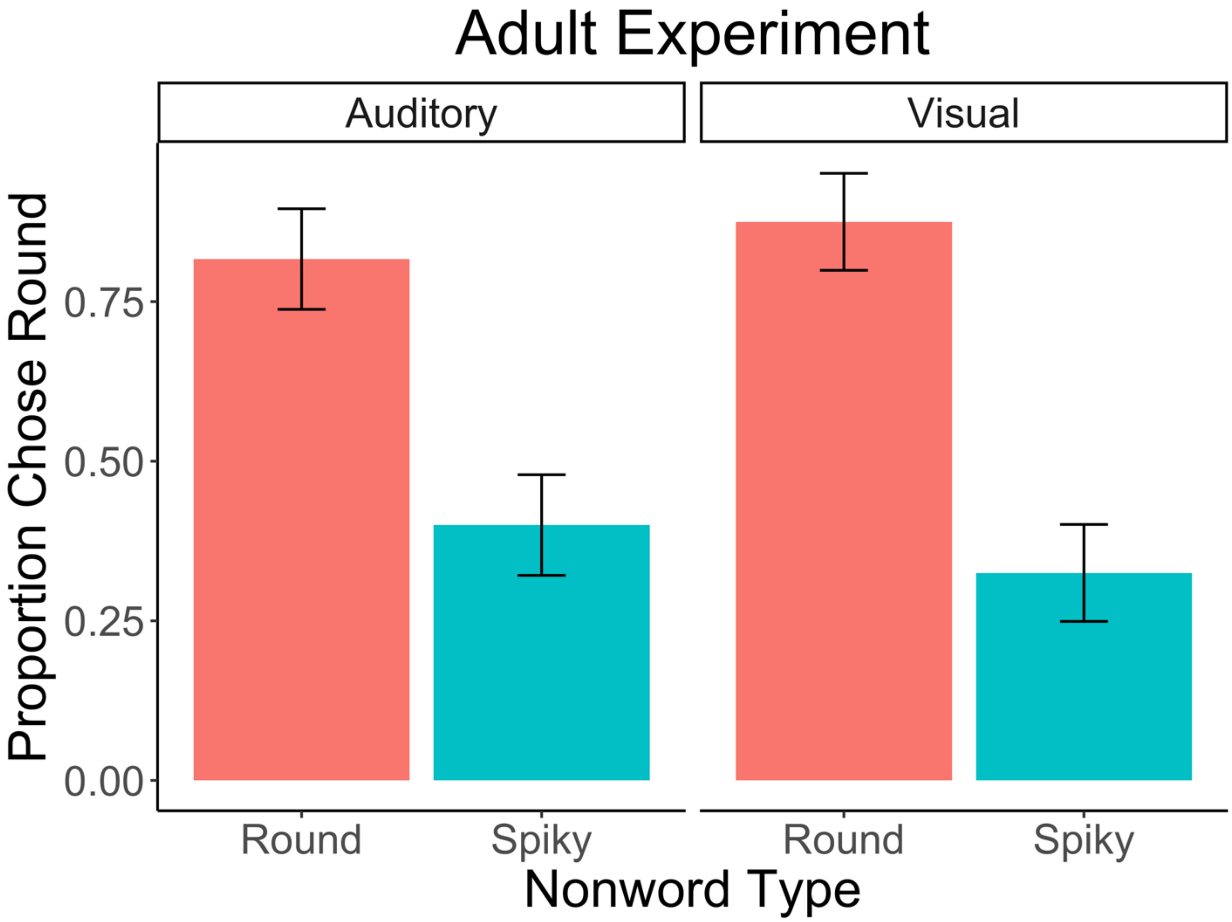

Note. This plot shows the proportion of trials on which adult participants chose the round shape as the best match for either type of nonword, in either the auditory (Experiment 1a) or visual (Experiment 1b) experiment. Error bars are 95% confidence intervals after applying the correction from Cousineau [38].

Best practice in data visualization recommends that every figure summarizing the results of more than one participant include the participant N on the plot (e.g., in the legend) or in the Figure description, so that a reader doesn't have to go searching for the info in other parts of the article.

Table 1

The Percent of Trials on Which Participants Paired Nonwords with Congruent Shapes in Experiments 1a and 1b

Table would be more informative if it included an indication of error/ variance about the % listed - e.g., 95% CIs

Nonword	Experiment 1a	Experiment 1b
/bobo/ - “boeboe”	81.67%	86.67%
/lulu/ - “looloo”	81.67%	88.33%
/keke/ - “kaykay”	61.67%	81.67%
/tʃitʃi/ - “cheechee”	58.33%	53.33%

Note. Nonwords are shown in their phonological form, and in their written form (presented in Experiment 1b).

Not sure what transliteration is being used by the team to describe the pronunciations. In the International Phonetic Alphabet the /o/ in /bobo/ is the monopthong vowel in 'got' NOT the dipthong vowel in 'go'. I think this might be the wrong symbol if the 'written label is 'boeboe'. If the word rhymes with 'logo' and 'oboe' then the symbol should be something like /ou/ or /oʊ/. Similar problem for the other vowels I think, but hard to be sure. This is a really common problem in bouba/kiki type research, and it makes replication and metaanalysis really hard. Please check your symbols align with the International Phonetic Alphabet, and/or to describe the stimulus words using unambiguous rhymes (e.g., 'oboe'). Where can a reader hear the audio tokens used in the study? I couldn't find them in the OSF repository.

Experiment 1b

Did the parents ever hear a member of the research team say the words aloud before the toys were sent home, or did they first encounter them written down? Was a parent's pronunciation checked to alignment to the expected wordform prior to in-lab test?

Method

Participants

Participants were 30 individuals (24 female; M_{Age} = 26.87, SD_{Age} = 6.29) recruited through the online platform Prolific (https://www.prolific.co/). Participants were paid at a rate of 7.50 GBP per hour. All participants reported English fluency and normal or corrected to normal vision.

Stimuli

The stimuli were the same as in Experiment 1a.

Procedure

The procedure was the same as in Experiment 1a, except that here nonwords were only presented visually onscreen in their written form instead of auditorily. The nonword appeared below the shape pair image.

Which written form? /bobo/ or "boeboe"?

Results

More detailed description needed - same as the first analysis (with random factor for participants?) or the second analysis?

This analysis was the same as that in Experiment 1a. The logistic regression revealed a significant effect of nonword type such that participants were 57.75 times more likely to choose the round shape for a round vs. a spiky nonword (b = 4.06, p < .001). See Figure 3. Participants chose the sound symbolically congruent shape on 78% of trials. A follow up analysis suggested that congruent choices were made significantly more often for round (88%) vs spiky (68%) nonwords (b = 1.22, p < .001). This difference was driven by results for the nonword *cheechee* which was only paired with a congruent shape 53% of the time (*boeboe* = 87%; *looloo* = 88%; *kaykay* = 82%), see Table 1. This rate of pairing for *cheechee* was lower than in Experiment 1a (58%) and could be due to its rounded orthography (see Bottini et al., 2019), a factor that will not play a role with infants in Experiments 2 and 3.

More detail needed (e.g., in supplement or code repository)

Discussion

We observed a maluma/takete effect with adult participants for our nonwords and visual stimuli. This is notable because the majority of sound symbolism studies have used simple two-dimensional shapes as stimuli (as illustrated in Figure 1), rather than the images of three-dimensional objects presented here.

NB. Shang and Styles (2017) uses 3D shapes

Experiment 2

Method

Participants

We recruited 63 infants (31 females) who were between the ages of four and five months (*M* = 4.87 months; *SD* = 0.54) at the time of their first testing session. They were tested again at 7-8 and 11-12 months. Infants were recruited from the University of Calgary's Child and Infant Learning and Development (Ch.I.L.D.) research group database. The infants received a t-shirt, a bath toy, and a certificate for participating in the experiment. All infants were exposed to English at least 50% of the time (3 who did not meet this criterion were excluded from analyses); 28 were exposed to another language (Cantonese, Spanish, Afrikaans, German, French, Russian, Hungarian, Dutch, Tagalog, Urdu, ASL, Swahili, Italian, German, Mandarin). Parents reported infants' ethnic background: 2 Asian; 42 White; 1 Black; 1 South Asian; 1 Arabic; 12 Mixed Ethnicity. Most parents (68%) had a university degree or higher. One infant was preterm, and their age was corrected to full term. There were two pairs of twin siblings.

Great level of detail in demographics! Are the full data in the archive?

Stimuli

The experimental stimuli consisted of the same visual and auditory nonword stimuli as in Experiment 1a. The materials for this experiment included a Developmental Questionnaire with 40 questions about children's physical, language, and communicative abilities that caregivers were asked to fill out weekly throughout the study in Qualtrics (Qualtrics, Provo, UT). A copy of this survey can be found here: https://osf.io/djgfx. Great use of Open Archiving :)

There were three components to the questionnaire. The first component was a set of questions about children's physical milestones (e.g., standing, walking, etc.). The caregivers

were asked to indicate their child's mastery of each milestone (9 in total) using a six point scale ("not yet", "just starting", "gaining confidence", "showing confidence", "doing moderately well", "mastered"). The second component was a set of questions about babbling of specific speech sounds. We included the speech sounds used in our nonwords and also speech sounds that are typically acquired earlier or are frequent in English.[2] For each speech sound (29 in total), the caregiver was asked to indicate the extent to which their child was producing the sound when they were babbling on a five point scale ("not yet", "maybe", "probably yes", "definitely yes", "frequently"). The third component was a set of questions about vocabulary development. Here the caregivers were asked to indicate for each of the 10 words that children commonly develop first (mom, dad, baba, yumyum, uhoh, bye, hi, grrr, woof, meow) whether their child understands the word on a five point scale ("not yet", "maybe", "probably yes", "definitely yes", "frequently").

Not clear whether this developmental survey was intended for exploratory analysis of the results of the current study, or simply to characterize the kids in the study according to their general development. Alternatively, these data might have been collected for use in a different/larger study. Please clarify.

When the child reached the age of 7-8 months (i.e., after their second lab visit), the set of questions for vocabulary slightly changed in that the caregivers were only asked to report any protowords the children might be producing and whether and how often they use Baby Sign with their child. The caregivers reported vocabulary development with the MacArthur-Bates Communicative Development Inventories Short-Form Level 1 [40] at the second and third visit (see below), replacing the 3 sets of questions described above. We also included another set of questions about the children's communicative development. From 7-8 months onwards the parents were asked to indicate whether children show an understanding of the communicative intent of others (e.g., responds to their name), whether they express communicative intent (e.g.,

It looks like only one version of the survey is currently in the archive - it would be great to see the full set there :)

[2] Full list of phonemes (described as "speech sounds") in the survey: /p, t, k, b, d, g, m, n, ŋ, f, s, v, z, w, j, l, ɹ, ʧ, i, u, e, o, ɪ, ʊ, ɛ, æ, ɑ, aɪ, aʊ/

extends arm to show what they hold, waves “bye-bye”, etc.), and whether they were using gestures to communicate (pointing, clapping, or other), on a three point scale (“not yet”, “sometimes”, “often”).

Many of the questions in the survey are not reported in the current writeup. There can be many reasons for not reporting all outcome measures, including that the bouba/kiki study reported here was part of a larger research project, which will be reported in full elsewhere. It is important to disclose the purpose fro collecting tis data so a reader can know whether there was exploratory analysis (i.e., multiple possible outcome measures were collected and only significant ones are reported in the current paper) or pre-conceived hypothesis tests. If exploratory, the analysis should be reported as such, along with the results of all non-significant tests, so that the probability values can be understood in context. If the study was preregistered, please clarify this in the main text of the study description so that the prereg can be readily identified.

Procedure

Infants were brought into the lab by their caregiver three times during the course of the experiment: at 4-5 months for their first visit, at 7-8 months for their second visit, and at 11-12 months for their third visit.

At the start of the first visit, while the infant familiarized themselves with their new surroundings, the caregiver and research assistant went over the consent process to confirm participation, filled out a demographics questionnaire, and a language questionnaire to determine the nature of the infant’s language environment. Once this was completed, the caregiver and infant were seated in the testing room (sound attenuated) to begin the study.

Are these questionnaires and the resulting data archived?

At each visit, to test infant sensitivity to sound symbolism, we used the Preferential Looking paradigm [e.g., 41]. Infants were seated on their caregiver’s lap or in a highchair with their caregiver sitting next to them. Infants faced a large smart-board screen (50 inches away from the infant) where the visual stimuli were shown. Caregivers were given noise cancelling headphones with music playing to prevent any influence on the infants’ looking patterns. The study was run using the Habit X 1.0 software (Cohen et al, 2004), and the infants’ looks were recorded using a camera (Canon VIXIA HF R600) positioned discreetly underneath the screen. Before every trial (and before the pre-test), a colorful bouncing ball appeared on the screen to attract the infant’s attention. The duration of the bouncing ball was controlled by the experimenter and stayed on the screen until the infant turned their attention to the screen (typically 3-4 s). Next, we presented infants with a 20-second pre-test (spinning pinwheel with

What is the degree of visual angle subtended by the two stimuli? this is important to understand as it may mean that children need to make just a saccade or a head movement to switch from one image to the other. This becomes important when considering the duration of looks included/discarded.

Is the code archived?

music) to further capture their attention to the screen. Following the pre-test, the test trials were presented, where infants saw the visual stimuli (pair of objects) accompanied with the auditory nonword (a single label repeated 12 times), which was kept at a range of 65 to 70dB. The entire trial lasted 15 seconds (ISI 151ms), unless the child did not attend for two seconds in which case it was terminated. Each infant was presented with 8 of these randomized trials, hearing each nonword twice.

During these test trials, on the rare occasion that an infant did not attend for a minimum of two seconds, the bouncing ball and pre-test were repeated to recapture the infant’s attention. After the test trials, we presented a post-test, which was identical to the pre-test (i.e., a spinning pinwheel with music for 20 seconds).

After the first visit, caregivers were sent weekly the Development Questionnaire using Qualtrics (Qualtrics, Provo, UT) until they were scheduled to come in for their second visit at 7-8 months-of-age.

During the second visit, parents filled out the MacArthur-Bates Communicative

Are the results from the CDI archived? (full data best, but score for each child also valuable if full data is embargoed for another study)

Development Inventories Short-Form Level 1 [40] to gain more information on the infants’ language understanding. Following this, infants were again presented with the preferential looking task. After the visit and weekly thereafter families were sent the Development Questionnaire, until they came in for their final visit when their infant reached the age of 11-12 months.

The final visit was identical to the second visit. Parents once again filled out the MacArthur-Bates Communicative Development Inventories Short-Form Level 1 [40], to track language development, and the infants were presented with the preferential looking task. A total of 48 infants completed all three visits.

Results

I'd like to hear more about your reasoning for this, and whether this was an a priori decision made data-blind (following an established protocol from previous study, before seeing current data), a condition-blind decision (applied after screening the data in a format where the condition of each trial was blind to the researcher making the decision), a results blind decision (made after a screening process where the condition of each trial was known, but before any between condition statistical comparison had been conducted), or a post-hoc decision after a preliminary analysis was conducted.

For each trial, we first calculated the amount of time spent looking at one of the two shapes onscreen (i.e., *looking time*). We excluded trials with an overall looking time of less than two seconds (1.56% of trials). We then calculated the percentage of looking time that was spent looking at the round object. Because participants could only look at the round or the spiky object, looking time to the round object was redundant with the percentage of looking time spent looking at the spiky object. Thus, 50% is the chance level, looking time above 50% indicates that infants are looking more to the round than the spiky object, and looking time below 50% indicates that infants are looking more to the spiky than the round object.

Perhaps I am missing something about the structure of the model, but without inclusion of a random factor for individual, this treats every visit as though it is a different group of children, and there is no repeated measures structure in the analysis - am I right? RM analyses usually have more power to detect within subjects effects.

Analyses consisted of linear mixed effects regressions. Our dependent variable was percent of looking time to the round object. We centered this variable by subtracting 0.5 in order to allow interpretation of the intercept. In every model, we fit fixed effects corresponding to the experimental manipulations of interest [37]. Our first set of analyses included: nonword type (using effects coding; round [+.5] vs. spiky [-.5]), visit number (using subsequent coding; i.e., comparing each visit to the one that came before), as well as their interaction, as predictors. Table 2 summarizes the results. The intercept was significantly less than 0 (b = -0.07, p < .001), indicating that participants spent longer looking at the spiky shape overall.[3] Despite matching round and spiky image pairs in terms of ratings of visual interest, it may have been that the spiky

Please refer your reader to Table for full statistical report

images were more interesting. We found a non-significant main effect of nonword type (p = .43),

[3] We also ran a control version of this task that did not include an auditory stimulus. Our motivation was to investigate if participants would still spend longer looking at the spiky object in the absence of any nonword. The participants were a separate sample of 4-month-olds (n = 20). There was indeed a bias to look at the spiky shape, with participants looking at the round shape for 46.06% of the trial (p = .04). However, this was significantly reduced compared to the 4-month-old group in Experiment 2 (40.67%; p = .03). Thus, despite our efforts to match shape pairs for visual interest using adult ratings, young infants tended to prefer to look at the spiky shapes. This suggests that infants did, at minimum, detect a visual difference between the two shapes.

which means that the looking time to the round object when a round nonword was played was not statistically different from when a spiky nonword was played. See Figure 4. There was also a non-significant main effect of visit three (vs. visit two $p = .71$), indicating that the looking time to the round object in visit three was not statistically different from that in visit two. There was however a significant main effect of visit two (vs. one; $b = 0.03$, $p = .03$), such that infants at visit two spent a greater percentage of time looking at the round shape than they did in visit one. Finally, no significant interaction between nonword type and visit was found (all *p*'s > .33). We also ran a supplementary analysis to examine if phoneme type (using effects coding; early [-.5] vs. late [+.5]) interacted with any of these predictors: it did not (all *p*'s > .39).

Table 2 should be mentioned in the text so a reader knows where to look for the full statistical output

Table 2

Linear Mixed Effects Regression Model Predicting Looking Time for All Infants With Nonword Type and Categorical Visit

Please add effect size for all effects including n.s. ones as this allows for future metaanalyses

Fixed Effect	B	SE	t	p
Intercept	-0.07	0.01	12.33	< .001***
Nonword Type	0.01	0.01	0.78	0.43
Visit (2 vs 1)	0.03	0.01	2.15	0.03*
Visit (3 vs 2)	0.01	0.01	0.37	0.71
Nonword Type x Visit (2 vs 1)	0.01	0.03	0.19	0.85
Nonword Type x Visit (3 vs 2)	0.01	0.03	0.97	0.34
Random Effect			s^2	
Participant Intercept			0.0003	

Figure 4

Percent Round Object Looking Time by Age and Nonword Type in Experiment 2

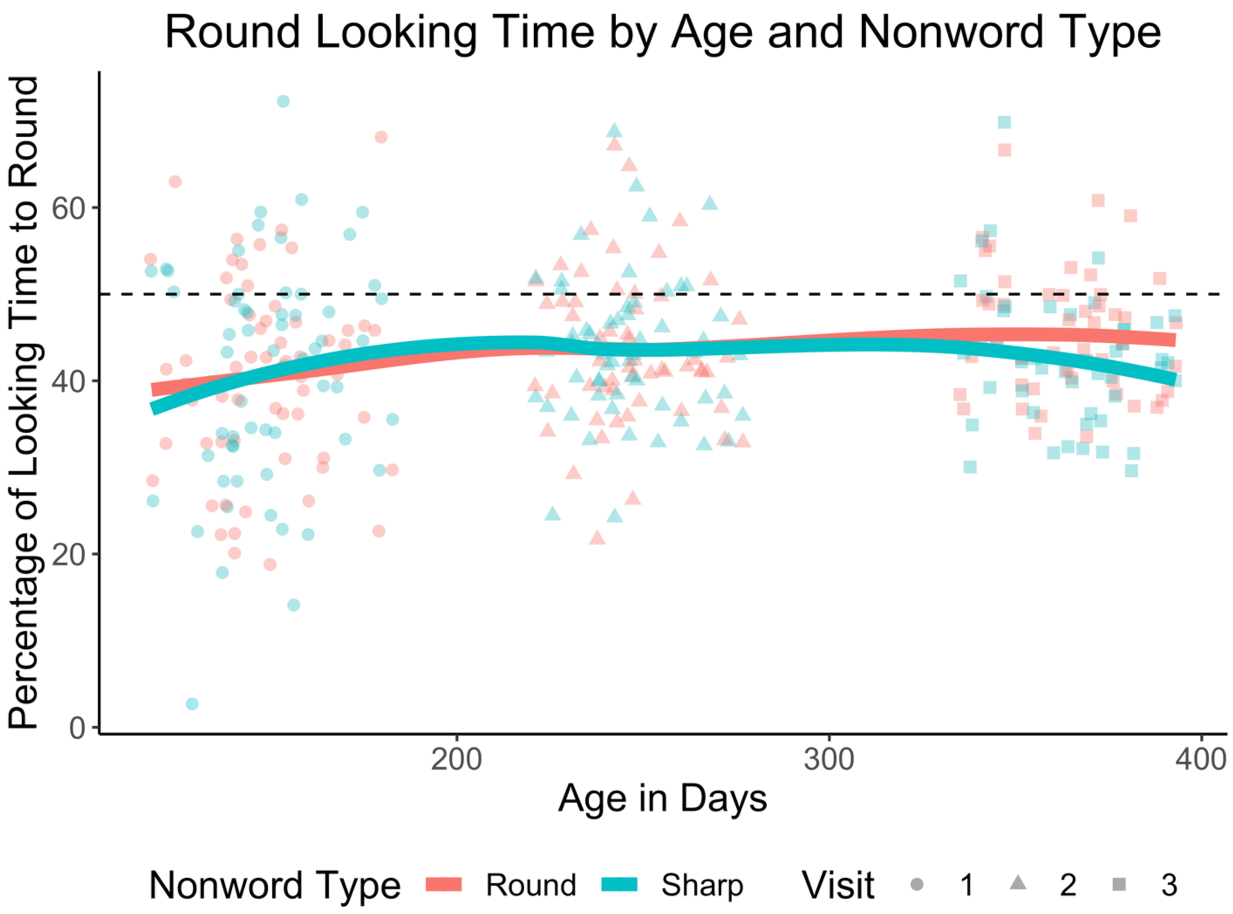

Note. This plot shows the percentage of total looking time spent looking at the round object, based on the age of the infant in days, and the type of nonword presented on each trial. The shape of each point corresponds to the visit number. Lines are LOESS functions. The dashed line corresponds to equal looking time between round and spiky shapes.

Please include sample size on figure, in Title or Note

In order to quantify the evidence in favour of the null hypotheses ~~reported above~~, we conducted a version of the analyses using Bayesian mixed effects regression. A full introduction to this approach is beyond the scope of this paper and we refer the reader to Vasishth et al. (2018) for a tutorial. We conducted a region of practical equivalence (*ROPE*) analysis (see Kruschke, 2018). This method examines the percentage of the highest density interval (*HDI*; i.e., the interval of credible values for each parameter) that falls within a *region of practical equivalence*. That is, instead of treating the null hypothesis as a point value of 0, it treats a range of values around 0, that are practically equivalent to 0, as representing the null. Here we adopt Kruschke's (2018) suggestion of a range of +/– 0.1 standard deviations in the dependent variable as the ROPE. This equates to what Cohen [42] considered a negligible effect size. We used the packages "rstanarm" [2.21.1] [43] and "bayestestR" [0.7.5] [44] to conduct Bayesian analyses.[4] When examining the interaction between nonword type and visit, only 36.93% (Visit 3 vs 2) and 55.05% (Visit 2 vs 1) of each HDI fell within the ROPE. However, 82.62% of the HDI for nonword type fell within the ROPE.

Please unpack this a little for the reader - what does this mean for interpretation of the null hypothesis?

We next ran a set of analyses that treated age as a continuous variable (after being standardized). Table 3 summarizes the results. We found again a non-significant main effect of nonword type (p = .49) and a non-significant interaction between nonword type and age (p = .30).[5] There was however a significant main effect of age (b = 0.01, p = .037) such that older children spent a greater percentage of time looking at the round shape than did the younger

[4] Models were run with 2000 iterations, the first 1000 of which were treated as a warm up. Four chains were run. If these models did not show good convergence (i.e., an effective sample size of at least 10% that of the total number of samples; [45]) additional iterations were added. Fixed effects were given a generic normal prior centered at 0, with a standard deviation of 2.5.

[5] Given the smaller effect size for the nonword *cheechee* in Experiments 1a and 1b, we ran versions of these analysis excluding trials with this item. The effect of nonword type, as well as its interaction with age (whether treated as a categorical or a continuous variable), were all nonsignificant (p's > .23). Despite a singular fit we included a random intercept for participant to account for nonindependence.

children.[6] A Bayesian analysis revealed that 79.40% of the HDI for the interaction between nonword type and age fell in the ROPE, as did 86.72% of the HDI for nonword type. We also ran again a supplementary analysis examining if phoneme type interacted with any of these predictors: it did not (all *p*'s > .35).

Table 3

Linear Mixed Effects Regression Model Predicting Looking Time for all Infants With Nonword Type and Age as a Continuous Variable

Fixed Effect	b	SE	t	p
(Intercept)	-0.07	0.01	12.52	< .001***
Nonword Type	0.01	0.01	0.69	.49
Age	0.01	0.01	2.15	0.04*
Nonword Type x Age	0.01	0.01	1.03	0.30
Random Effect		s^2		
Participant Intercept		0.0003		
Participant Age Slope		0.0005		

We proceeded with planned comparisons at each visit separately. These analyses only included nonword type as a predictor. They revealed again a non-significant effect of nonword

[6] Here and in the analysis with age as a categorical predictor, the pattern of results do not change when only including children who attended all three sessions (*n* = 48).

Please include all analyses - including non-significant ones - in the archive for the article or supplementary materials.

type at visits one (p = .87), two (p = .93) and three (p = .09).[7] Bayesian analyses revealed that 73.45%, 71.85%, and 24.27% of the HDIs for nonword type, respectively, fell within the ROPE.

Individual Differences

Because there was a trend towards sensitivity to sound symbolism developing at visit three, we focused on individual difference measures from that visit. For each Developmental Questionnaire item, the answers were dichotomozied: 0 = child does not have a given ability; 1 = child has the given ability. A full description of this coding can be found at: https://osf.io/djgfx. Our goal was to operationalize various potential developmental milestones: experience producing speech sounds, language knowledge and environmental experience. To operationalize experience producing speech sounds, we performed an exploratory factor analysis on babbling

at what timepoint? this is not included at timepoint 1, and I wasn't able to find a version including these questions in the archive

data. Because these data were binary, we performed factor analysis using tetrachoric correlations. We began with babbling scores for the eight phonemes used in our study (i.e., /b, l, k, tʃ, o, u, i, e/). However, we excluded /b/ because all babies were babbling this speech sound, as well as the phonemes /u/ and /i/ because most babies were babbling these speech sounds (> 90%).[8] A parallel analysis suggested reducing these variables to a single factor. We operationalized language knowledge as the understanding and productive scores on the MCDI. Finally, we operationalized environmental experience by performing an exploratory factor analysis on the following physical milestones: walking with support, walking alone, and going up and down stairs. Parallel analysis once again suggested a single factor. This created five

First mention of this in this section - also derived from factor analyses, or a raw score from original survey?

individual difference measures: a babbling factor, a vocal play score, an MCDI understanding score, an MCDI production score, and an environmental experience component.

Full statistical reports for the factor analyses not yet in the Archive?
Please add a supplement

[7] These were all nonsignificant when removing *cheechee* trials (p's > .15). Despite a singular fit for visits one and two, we included a random intercept for participant to account for nonindependence.

[8] Including these measures in the subsequent factor analysis leads to a non-positive definite correlation matrix.

unclear reasoning here... I understand that there were different surveys at different time points, but am I correct that only visit 3 is included in this section of the paper? Both for the survey and for the test data? It would be great to hear more about the reasoning for only testing T3.

Next, we operationalized sensitivity to sound symbolism at visit three as the average percent looking time to the congruent shape across both round and spiky trials. We also calculated the change in this variable from visit two to visit three, to quantify an increase in the sensitivity to sound symbolism. See Table 4 for a summary of all variables. We then computed eight correlations, one for each of these outcome variables and our individual differences, see Table 5. Only the correlation between the babbling factor and sensitivity to sound symbolism at visit three was significant (r = .31, p = .045), such that those who babbled more of the speech sounds from our nonword stimuli showed a larger effect. See Figure 5. However, this

Important to note in an exploratory analysis of this kind :)

relationship would not survive correction for multiple comparisons. It is also worth noting that the babbling factor was marginally correlated with the increase in sound symbolism from visits two and three (r = .28, p = .08).

not significant if the alpha was .05. Best to avoid the expression 'marginal' if you are doing null hypothesis statistical testing as it makes statisticians really mad.

Perhaps I wasn't following clearly, but I wasn't sure how time was implemented for each of these correlations. The surveys were conducted at 3 time points, as were the gaze direction tests. Were the factor analyses run separately for each time point and then correlations were tested only for synchronous correspondence between survey and test? Or wwere all score across time points collapsed into a single measure of individual difference?

Table 4

The data collected at all 3 timepoints is interesting to other resaerchers in the field so unless reserved for another study, it would be better to report values for all survey data at all time poitns if possible.

Summary Statistics of Individual Difference Measures at Visit Three

Variable	Percentage or Mean (SD)
Percentage Babbling /b/	100%
Percentage Babbling /l/	53.66%
Percentage Babbling /k/	52.8%
Percentage Babbling /tʃ/	39.2%
Percentage Babbling /o/	80.95%
Percentage Babbling /u/	92.86%
Percentage Babbling /i/	90.8%
Percentage Babbling /e/	85.1%
Percentage Walking with Support	76.19%
Percentage Walking Alone Easily	24.39%
Percentage Traversing Stairs	61.90%
MCDI Understanding out of 89	16.73 (10.57)
MCDI Production out of 89	1.34 (1.74)

This text is unlcear. Perhaps better to split into two tables, or two columns in one table. One for table/column could be for binary milestones, where the number reported is % of children, and one table/column where the number is the mean score.

Might be nice to present these data together with some indication of error/variance e.g., Confidence Intervals

Sound Symbolism Effect (Look Percentage to Congruent Minus Incongruent)	1.47 (4.09)
Change in Effect Visit 2 to Visit 3	1.18 (5.72)

Table 5

Where are the mean scores for the EFA reported/Archived?

Correlations Between Individual Difference Measures at Visit Three and Sound Symbolism Measures

	Sound Symbolism Sensitivity at Visit Three<br>*r* (*p*)	Change from Visit Two to Visit Three<br>*r* (*p*)
Babbling Factor	.31 (.045)	.28 (.08)
Vocal Play	.02 (.90)	-.00 (.99)
MCDI Understanding	-.14 (.37)	-.03 (.84)
MCDI Production	-.01 (.96)	-.14 (.39)
Movement Factor	.19 (.23)	-.00 (.98)

Figure 5

Correlation Between Babbling Factor Score and Percent Looking Time to Congruent Shape at Visit 3

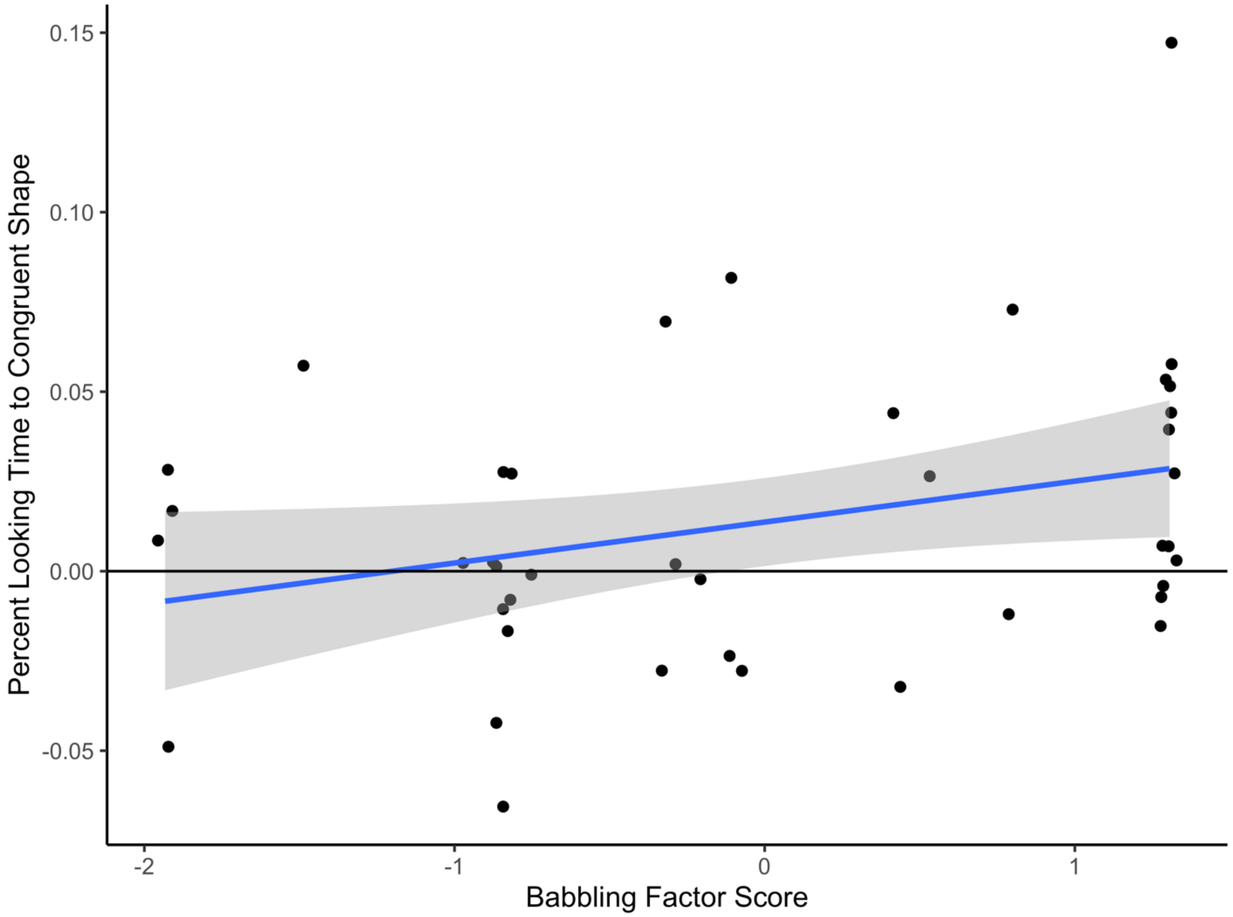

Discussion

We did not find a maluma/takete effect in our sample as a whole; nor at 4, 8 or 12 months of age. There was a trend towards this effect emerging at 12 months, however it was not significant. We found a moderate correlation between babbling experience and sensitivity to sound symbolism at 12 months, but this would not survive correction for multiple comparisons.

Experiment 3

Methods

Participants

We recruited a separate group of 71 infants ages 7-8 months (M = 7.89; SD = 0.30) again from the Ch.I.L.D. participant database at the University of Calgary and with a minimum 50% exposure to English; 26 were exposed to another language (Mandarin, Russian, Cantonese, Ukrainian, Japanese, French, Spanish, Farsi, Yoruba, Polish, Czech, German, Punjabi). Of these infants, 11 were excluded due to technology error, fussiness, mistake in label pronunciation, dropping out of the study, insufficient English exposure, or being too old for the study by the time they were tested. The target sample size was 30 infants per condition, with 15 per congruent and incongruent labels.

Materials

In the preferential looking task we used the stimuli from Experiment 2 and we also included two new items. This consisted of a new round nonword (/mumu/) and spiky nonword (/titi/). Each of these was presented twice in the task during the second visit.

Procedure

The procedure was similar to that described for Experiment 2, except that here families came for two visits about two weeks apart, and completed a learning task in between visits. During the first visit, caregivers completed the demographic and language measures and the infant completed the preferential looking task used in Experiment 2. At the end of the first visit, families were given a learning task to complete with their infant. That is, they were given a 3D object (Figure 6; either round or angular, and in one of the two colours, with a string attached) to

take home. Families were instructed to expose their infant to the object for 10 minutes a day, saying the label a minimum of 12 times, for 14 days. Caregivers were instructed to not leave the infant alone with the object to ensure that the child’s experience with the object always involved exposure to the label.

Figure 6

3D Printed Objects that Families Took Home

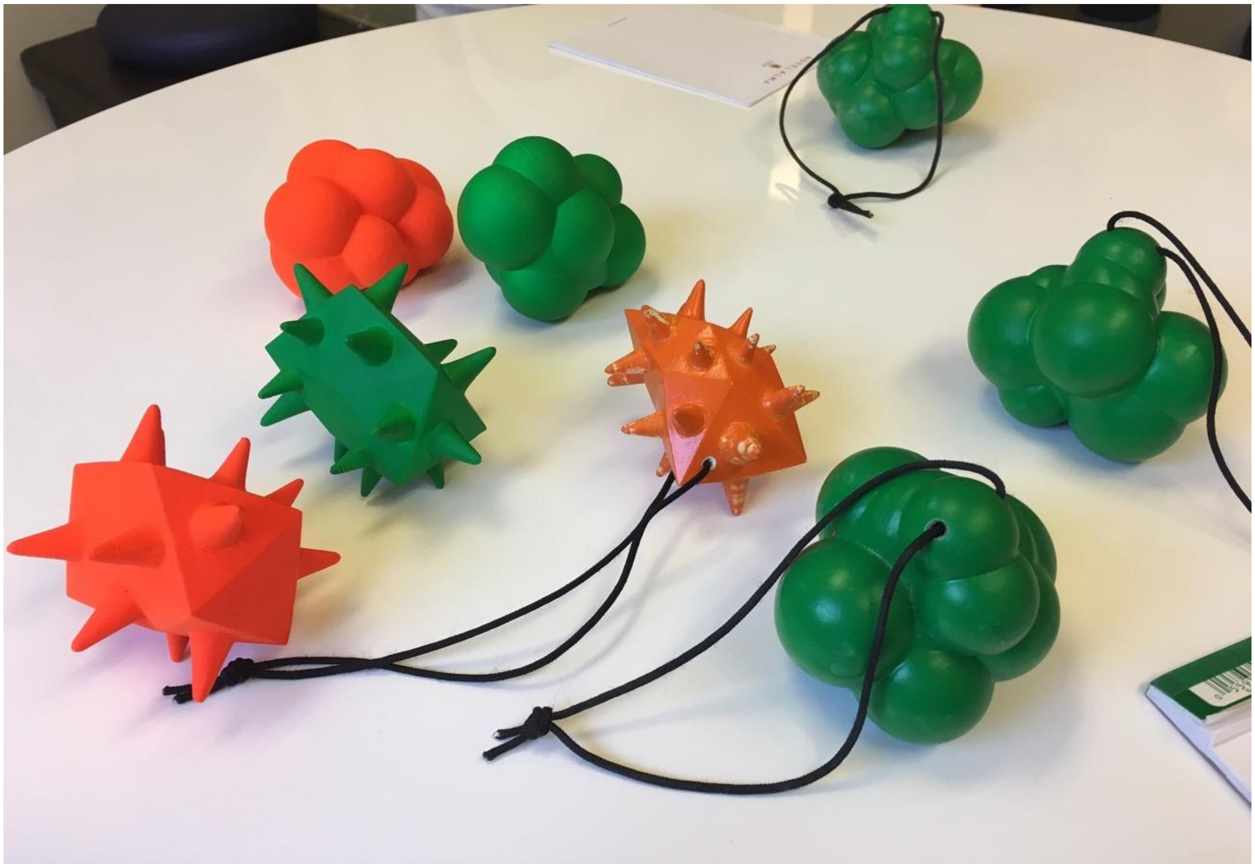

Note. All finished objects had strings attached.

Families returned to the lab approximately two weeks after their first visit, give or take one or two days. During this second visit, a research assistant asked caregivers about how their learning task went, checking whether the instructions were followed and asking about the way they interacted with their infant during the learning task. Next, families were once again seated in our testing room. Testing conditions were identical to the first visit except that the second visit included four extra trials with novel nonwords (i.e., /mumu/ and /titi/). These additional trials allowed us to test whether infants learned the sound-symbolism bias and could apply it to novel labels as well as familiar ones.

Results

Analyses consisted of linear mixed effects regressions. Our dependent variable was percent of looking time to the congruent object. We centered this variable by subtracting 0.5 in order to allow interpretation of the intercept. Our initial analysis included: visit (after training [+.5] vs. before training [-.5]), training congruence (congruent [.5] vs. incongruent [-.5]), as well as their interaction, as predictors. Table 6 summarizes the results. The intercept was not significantly different from 0 (b = 0.00, p = .99), indicating that participants did not show a preference for the congruent shape overall. We found non-significant effects of visit (b = -0.01, p = .10) and training congruence (b = 0.02, p = .15). The interaction between predictors was not significant (b = 0.00, p = .80).[9] A Bayesian analysis revealed that 91.45% of the HDI for the interaction between visit and training congruence fell in the ROPE.

Need some data display to contextualize the results relative to the previous study (even if non significant

[9] We ran a version of this analysis excluding trials with the nonword "cheechee". The effect of visit as well as its interaction with congruence were both nonsignificant (p's > .12).

Table 6

Linear Mixed Effects Regression Model Predicting Looking Time for All Infants with Visit, Training Congruence, and their Interaction

effect size?

Fixed Effect	b	SE	t	p
Intercept	-0.00	0.03	-0.003	.99
Visit	-0.00	0.00	-1.66	.10
Training Congruence	0.02	0.01	1.45	.15
Visit x Training Congruence	-0.00	0.01	-0.26	.80
**Random Effect**		s^2		
Item Intercept		0.01		

We conducted a supplementary analysis to examine whether participants showed any evidence of learning the trained nonword. This analysis used the trials on which participants heard the nonword that they had received in training, and used the percent of looking time to the trained shape as the dependent variable. We used visit as the predictor of interest. Visit was not a significant predictor (*b* = -0.00, *p* = .74).[10] A Bayesian analysis revealed that 86.25% of the HDI for Visit fell in the ROPE. This suggests that participants did not learn the nonword-shape pairing. We also examined if participants looked longer at the screen on trials where the colour of the shapes matched the trained shape and found that they did not (*b* = -13.04, *p* = .64). A

[10] This was also non-significant when only including trials that matched the colour of the object infants took home (*p* = .90).

Bayesian analysis revealed that 85.5% of the HDI for the familiar colour predictor fell in the ROPE.[11]

Discussion

We did not find evidence that experience with a sound symbolically congruent label affected sensitivity to sound symbolism. In fact, we found that infants were more sensitive to sound symbolism *before* any training took place. Nevertheless, we should be cautious when interpreting the results from this study, as there was no evidence of training having an effect (no indication of greater attention to the take home object when hearing the trained nonword).

General Discussion

After verifying that the maluma/takete effect could be observed in adults using our novel shape stimuli (Experiments 1a and 1b), we conducted the first longitudinal study of infant sound symbolism (Experiment 2). This consisted of a looking time preference task comparing attention to a round and spiky shape while infants heard either a round- or a spiky-sounding nonword. Infants were tested at 4, 8 and 12 months. We found no evidence of sensitivity to sound symbolism: no difference in infants' attention to round and spiky shapes based on auditory stimuli. This was true overall, and also when the data from each visit were analyzed separately. There was, however, a trend towards sensitivity at 12 months. In addition, a factor representing individual babbling ability was moderately correlated with this effect. A subsequent study (Experiment 3) in which parents presented 8-month-old infants with congruent or incongruent labels for 3-D printed shapes also found no evidence of sensitivity to sound symbolism following training, for either label condition.

[11] Note that in both of these instances we were able to include a random intercept for family in the Bayesian analysis, but not the frequentist model.

The first conclusion of note is that sensitivity to sound symbolism does not appear to be innate. This is consistent with previous work which found no evidence of sound symbolism in four months old infants [27]. Some researchers have suggested that the neonatal brain contains widespread connectivity among sensory regions, which then become pruned during development (reviewed in [31]). This theory would predict sensitivity to crossmodal associations like the maluma/takete effect from very early on. The present results do not support this. Our inference that shape sound symbolism is late-emerging is in contrast with other, non-linguistic, crossmodal associations which have been observed at a young age: between visual elevation and sharpness at 3-4 months [46], between pitch and size at 6 months (but not 4 months; [47]), and between loudness and brightness at 20-30 days [48]. Perhaps the involvement of the linguistic system in sound symbolism leads to a slightly different etiology than that of other crossmodal associations (see [21]; cf. [49]).

See also the dissertation by Woon 2018 where 9-year olds show less tendency for sound symbolic responding than adults

Although we only observed marginal sensitivity to sound symbolism at 12 months, other studies have found an effect in infants of that age [11,27,28]. If this is indeed the age by which sound symbolism emerges, what factors might contribute to its development? In the Introduction we proposed several possibilities. One of these was language experience. Fort et al. [31] discussed the possibility of language acting as a “revelator” of sound symbolism. That is, infants may be born with the sensitivity to sound symbolic relationships, but experience with sound symbolic words in language serves to “reveal” specific associations. However, we did not find any relationships between infants’ receptive or productive vocabulary and their sensitivity to sound symbolism. Further, exposure to sound symbolic language in Experiment 3 (i.e., those in the congruent label condition) did not increase this sensitivity.

Another possibility was that environmental experience may play a key role in the development of sound symbolism. This relies on the assumption that round and spiky objects in the world tend to make different sounds, and that these sounds mimic round- and spiky-associated speech sounds [4]. There is some tangential evidence for the role of environmental experience in sound symbolism. Work with visually- as well as hearing-impaired individuals suggests a sensitive period in which perceptual experience is key to the later development of sound symbolism [50,51]. One might posit that this perceptual experience comprises patterns in the environment. Yet we did not find any evidence that the ability to explore the environment was related to sound symbolic sensitivity. It is important to note, however, that this is a rather coarse measure of environmental experience. Even before they begin to move themselves, infants are moved through the world by caregivers, and have objects brought to them by caregivers. A more direct way to test this possibility could be to track an infant's daily experience in the environment (e.g., [52]).

Ohala was the first to propose physical relationships of this kind

We did find that babbling ability was related to sound symbolism. Infants who babbled a greater number of speech sounds tended to show a greater sensitivity to sound symbolism. This could suggest that articulatory experience is a key contributor to the effect. Indeed, the role of oral motor movements in early infant speech perception is only beginning to be understood (e.g., [53]). Choi et al. recently demonstrated that oral motor interference diminished speech discrimination in 3-month-old prebabbling infants. Infants who have more babbling experience may also have more oral motor experience and thus more sensitivity to the sound-shape associations involved in the maluma/takete effect. This could be tangentially related to the cross-cultural finding that the maluma/takete effect may not emerge when the speech sounds in a nonword are not present in a speaker's language, or are in phonotactically illegal positions [6].

See also Shang & Styles (2017, 2023)for differences in the nature of sound symbolism for langs with different acoustic features.

However, the correlation we observed should not be over-interpreted as it would not survive correction for multiple comparisons.

What other developmental milestones, not captured here, might explain the emergence of sound symbolism? It may be that some specific neurological development is necessary. Indeed, maturation of the association cortex (including areas implicated in sound symbolism; [28,30]) occurs later than individual sensory areas [54]. Work from individuals with diagnosis of Autism Spectrum Disorder and dyslexia has suggested that a key ability in sound symbolism is multisensory integration [55–57]—this could be another developmental milestone. Future work might also measure specific language milestones, such as the development of phonological awareness, referential insight[12], or the ability to process metaphor.

It is worth exploring why we did not observe a robust sound symbolism effect at 12 months when other studies have done so [11,27,28]. One relevant difference may be that each of these previous studies used a single image task, while we presented infants with pairs of images. Note that Fort et al. [58] also found no evidence of the maluma/takete effect in 5- and 6-month-olds, using a looking time preference task with pairs of images. This led them to speculate that very early sound symbolism may only be detectable with simpler designs (i.e., with a single presented image, re-using the same round and spiky images on each trial). In addition, we only observed a modest sound symbolic effect for our spiky nonwords/images with adult participants in Experiments 1a and 1b. Using pictures of real objects may have made the curves and spikes in

But see Bottini, Barilari & Collignon (2017), Shang & Styles (2017) for strong effects in 3D (albeit different)

our stimuli less salient than in the simple and more abstract figures typically used. Compare the outlines of the shapes in Figure 1 with those in Figure 2, for example. At minimum, we can

[12] As a way of examining this, we ran a supplementary analysis to see if a participant's looking time preference to the shape they were trained on while hearing their trained label at visit 2 of Experiment 3, predicted their sensitivity to sound symbolism, it did not (b = -0.01, p = .39).

It is also worth noting that there may be a large 'file drawer of unreported 'failed' studies within this age range, thereby masking whether the effectis robust and reliable at this early age.

conclude that infant sound symbolism is not an especially robust phenomenon, and that it is sensitive to methodological details, the specific influence of which is not yet well understood.

Conclusion

We did not find evidence of sound symbolism in infants at 4, 8 or 12 months of age. However, consistent with other work Perjoic and Molnar [27], there was a trend towards sound symbolism emerging at 12 months. This emerging sensitivity correlated with the development of babbling in individual infants, but not with their ability to explore the environment or their vocabulary. Further investigations of individual differences in the development of sound symbolism could be a fruitful area for future research.

Acknowledgements

This research was supported by the Social Sciences and Humanities Research Council of Canada (SSHRC) through an Insight Development Grant (430-2017-00003). The authors would like to thank Dr. Ford Burles for creating the 3D objects used in these studies, Christopher Engelking for helping with data management, and Summer Abdalla and Julia Kim for helping with data collection.

References

1. de Saussure F. Course in General Linguistics. New York, NY: Columbia University Press; 1916.
2. Dingemanse M, Blasi DE, Lupyan G, Christiansen MH, Monaghan P. Arbitrariness, Iconicity, and Systematicity in Language. Trends Cogn Sci. 2015;19: 603–615. doi:10.1016/j.tics.2015.07.013
3. Murgiano M, Motamedi Y, Vigliocco G. Situating Language in the Real-World: The Role of Multimodal Iconicity and Indexicality. J Cogn. 2021;4: 38. doi:10.5334/joc.113
4. Fort M, Schwartz J-L. Resolving the bouba-kiki effect enigma by rooting iconic sound symbolism in physical properties of round and spiky objects. Sci Rep. 2022;12: 19172. doi:10.1038/s41598-022-23623-w
5. Köhler W. Gestalt Psychology. New York, NY: Liverlight; 1929.
6. Styles SJ, Gawne L. When Does Maluma/Takete Fail? Two Key Failures and a Meta-Analysis Suggest That Phonology and Phonotactics Matter. -Percept. 2017;8: 2041669517724807. doi:10.1177/2041669517724807
7. McCormick K, Kim JY, List S, Nygaard LC. Sound to Meaning Mappings in the Bouba-Kiki Effect. Proceedings of the 37th Annual Conference of the Cognitive Science Society. Pasadena, CA; 2015.
8. Sidhu DM, Vigliocco G, Pexman PM. Higher order factors of sound symbolism. J Mem Lang. 2022;125: 104323. doi:10.1016/j.jml.2022.104323

9. Sidhu DM, Pexman PM. A Prime Example of the Maluma/Takete Effect? Testing for Sound Symbolic Priming. Cogn Sci. 2017;41: 1958–1987. doi:10.1111/cogs.12438

10. Parise CV, Spence C. Audiovisual crossmodal correspondences and sound symbolism: a study using the implicit association test. Exp Brain Res. 2012;220: 319–333. doi:10.1007/s00221-012-3140-6

11. Asano M, Imai M, Kita S, Kitajo K, Okada H, Thierry G. Sound symbolism scaffolds language development in preverbal infants. Cortex. 2015;63: 196–205. doi:10.1016/j.cortex.2014.08.025

12. Imai M, Kita S. The sound symbolism bootstrapping hypothesis for language acquisition and language evolution. Philos Trans R Soc B Biol Sci. 2014;369: 20130298. doi:10.1098/rstb.2013.0298

13. Nielsen AK, Dingemanse M. Iconicity in Word Learning and Beyond: A Critical Review. Lang Speech. 2021;64: 52–72. doi:10.1177/0023830920914339

14. Perry LK, Perlman M, Lupyan G. Iconicity in English and Spanish and Its Relation to Lexical Category and Age of Acquisition. PLOS ONE. 2015;10: e0137147. doi:10.1371/journal.pone.0137147

15. Sidhu DM, Williamson J, Slavova V, Pexman PM. An investigation of iconic language development in four datasets. J Child Lang. 2022;49: 382–396. doi:10.1017/S0305000921000040

16. Imai M, Kita S, Nagumo M, Okada H. Sound symbolism facilitates early verb learning. Cognition. 2008;109: 54–65. doi:10.1016/j.cognition.2008.07.015

17. Cuskley C, Simner J, Kirby S. Phonological and orthographic influences in the bouba–kiki effect. Psychol Res. 2017;81: 119–130. doi:10.1007/s00426-015-0709-2

18. Ćwiek A, Fuchs S, Draxler C, Asu EL, Dediu D, Hiovain K, et al. The *bouba/kiki* effect is robust across cultures and writing systems. Philos Trans R Soc B Biol Sci. 2022;377: 20200390. doi:10.1098/rstb.2020.0390

19. Knoeferle K, Li J, Maggioni E, Spence C. What drives sound symbolism? Different acoustic cues underlie sound-size and sound-shape mappings. Sci Rep. 2017;7: 5562. doi:10.1038/s41598-017-05965-y

20. Lacey S, Jamal Y, List SM, McCormick K, Sathian K, Nygaard LC. Stimulus Parameters Underlying Sound-Symbolic Mapping of Auditory Pseudowords to Visual Shapes. Cogn Sci. 2020;44: e12883. doi:10.1111/cogs.12883

21. Sidhu DM, Pexman PM. Five mechanisms of sound symbolic association. Psychon Bull Rev. 2018;25: 1619–1643. doi:10.3758/s13423-017-1361-1

22. Spence C. Crossmodal correspondences: A tutorial review. Atten Percept Psychophys. 2011;73: 971–995. doi:10.3758/s13414-010-0073-7

23. Taylor IK. Phonetic symbolism re-examined. Psychol Bull. 1963;60: 200–209. doi:10.1037/h0040632

24. Sidhu DM, Westbury C, Hollis G, Pexman PM. Sound symbolism shapes the English language: The maluma/takete effect in English nouns. Psychon Bull Rev. 2021;28: 1390–1398. doi:10.3758/s13423-021-01883-3

25. Maurer D, Pathman T, Mondloch CJ. The shape of boubas: sound-shape correspondences in toddlers and adults. Dev Sci. 2006;9: 316–322. doi:10.1111/j.1467-7687.2006.00495.x

26. Ozturk O, Krehm M, Vouloumanos A. Sound symbolism in infancy: Evidence for sound–shape cross-modal correspondences in 4-month-olds. J Exp Child Psychol. 2013;114: 173–186. doi:10.1016/j.jecp.2012.05.004

27. Pejovic J, Molnar M. The development of spontaneous sound-shape matching in monolingual and bilingual infants during the first year. Dev Psychol. 2017;53: 581–586. doi:10.1037/dev0000237

28. Yang J, Asano M, Kanazawa S, Yamaguchi MK, Imai M. Sound symbolism processing is lateralized to the right temporal region in the prelinguistic infant brain. Sci Rep. 2019;9: 13435. doi:10.1038/s41598-019-49917-0

29. Hashimoto T, Usui N, Taira M, Nose I, Haji T, Kojima S. The neural mechanism associated with the processing of onomatopoeic sounds. NeuroImage. 2006;31: 1762–1770. doi:10.1016/j.neuroimage.2006.02.019

30. Kanero J, Imai M, Okuda J, Okada H, Matsuda T. How Sound Symbolism Is Processed in the Brain: A Study on Japanese Mimetic Words. PLOS ONE. 2014;9: e97905. doi:10.1371/journal.pone.0097905

31. Fort M, Lammertink I, Peperkamp S, Guevara-Rukoz A, Fikkert P, Tsuji S. Symbouki: a meta-analysis on the emergence of sound symbolism in early language acquisition. Dev Sci. 2018;21: e12659. doi:10.1111/desc.12659

32. Crowe K, McLeod S. Children's English Consonant Acquisition in the United States: A Review. Am J Speech Lang Pathol. 2020;29: 2155–2169. doi:10.1044/2020_AJSLP-19-00168

33. Bates D, Mächler M, Bolker B, Walker S. Fitting Linear Mixed-Effects Models Using **lme4**. J Stat Softw. 2015;67. doi:10.18637/jss.v067.i01

34. Singmann H, Bolker B, Westfall J, Aust F. afex: Analysis of Factorial Experiments. 2016.

35. R Core Team. R: A language and environment for statistical computing. R Foundation for Statistical Computing. Vienna, Austria; 2021. Available: https://www.R-project.org/

36. Brauer M, Curtin JJ. Linear mixed-effects models and the analysis of nonindependent data: A unified framework to analyze categorical and continuous independent variables that vary within-subjects and/or within-items. Psychol Methods. 2018;23: 389–411. doi:10.1037/met0000159

37. Meteyard L, Davies RAI. Best practice guidance for linear mixed-effects models in psychological science. J Mem Lang. 2020;112: 104092. doi:10.1016/j.jml.2020.104092

38. Cousineau D. Confidence intervals in within-subject designs: A simpler solution to Loftus and Masson's method. Tutor Quant Methods Psychol. 2005;1. doi:10.20982/tqmp.01.1.p042

39. Bottini R, Barilari M, Collignon O. Sound symbolism in sighted and blind. The role of vision and orthography in sound-shape correspondences. Cognition. 2019;185: 62–70. doi:10.1016/j.cognition.2019.01.006

40. Fenson L, Dale PS, Reznick JS, Bates E, Thal DJ, Pethick SJ, et al. Variability in Early Communicative Development. Monogr Soc Res Child Dev. 1994;59: i–185. doi:10.2307/1166093

41. Golinkoff RM, Ma W, Song L, Hirsh-Pasek K. Twenty-Five Years Using the Intermodal Preferential Looking Paradigm to Study Language Acquisition: What Have We Learned? Perspect Psychol Sci. 2013;8: 316–339. doi:10.1177/1745691613484936

42. Jacob Cohen. Statistical power analysis for the behavioral sciences. 2ns ed. Hillsdale, NJ: Erlbaum; 1988.

43. Goodrich B, Gabry J, Ali I, Brilleman S. rstanarm: Bayesian applied regression modeling via Stan. 2020. Available: https://mc-stan.org/rstanarm

44. Makowski D, Ben-Shachar M, Lüdecke D. bayestestR: Describing Effects and their Uncertainty, Existence and Significance within the Bayesian Framework. J Open Source Softw. 2019;4: 1541. doi:10.21105/joss.01541

45. Vasishth S, Nicenboim B, Beckman ME, Li F, Kong EJ. Bayesian data analysis in the phonetic sciences: A tutorial introduction. J Phon. 2018;71: 147–161. doi:10.1016/j.wocn.2018.07.008

46. Walker P, Bremner JG, Mason U, Spring J, Mattock K, Slater A, et al. Preverbal Infants' Sensitivity to Synaesthetic Cross-Modality Correspondences. Psychol Sci. 2010;21: 21–25. doi:10.1177/0956797609354734

47. Fernández-Prieto I, Navarra J, Pons F. How big is this sound? Crossmodal association between pitch and size in infants. Infant Behav Dev. 2015;38: 77–81. doi:10.1016/j.infbeh.2014.12.008

48. Lewkowicz DJ, Turkewitz G. Cross-modal equivalence in early infancy: Auditory–visual intensity matching. Dev Psychol. 1980;16: 597–607. doi:10.1037/0012-1649.16.6.597

49. Peña M, Mehler J, Nespor M. The Role of Audiovisual Processing in Early Conceptual Development. Psychol Sci. 2011;22: 1419–1421. doi:10.1177/0956797611421791

50. Gold R, Segal O. The Bouba–Kiki Effect in Persons with Prelingual Auditory Deprivation. Lang Learn Dev. 2020;16: 49–60. doi:10.1080/15475441.2019.1685386

51. Sourav S, Kekunnaya R, Shareef I, Banerjee S, Bottari D, Röder B. A Protracted Sensitive Period Regulates the Development of Cross-Modal Sound–Shape Associations in Humans. Psychol Sci. 2019;30: 1473–1482. doi:10.1177/0956797619866625

52. Slone LK, Smith LB, Yu C. Self-generated variability in object images predicts vocabulary growth. Dev Sci. 2019;22: e12816. doi:10.1111/desc.12816

53. Choi D, Dehaene-Lambertz G, Peña M, Werker JF. Neural indicators of articulator-specific sensorimotor influences on infant speech perception. Proc Natl Acad Sci. 2021;118: e2025043118. doi:10.1073/pnas.2025043118

54. Gogtay N, Giedd JN, Lusk L, Hayashi KM, Greenstein D, Vaituzis AC, et al. Dynamic mapping of human cortical development during childhood through early adulthood. Proc Natl Acad Sci. 2004;101: 8174–8179. doi:10.1073/pnas.0402680101

55. Drijvers L, Zaadnoordijk L, Dingemanse M. Sound-Symbolism is Disrupted in Dyslexia: Implications for the Role of Cross-Modal Abstraction Processes. Proceedings of the 37th Annual Meeting of the Cognitive Science Society. Austin, TX; 2015. p. 6.

56. Gold R, Segal O. The bouba-kiki effect and its relation to the Autism Quotient (AQ) in autistic adolescents. Res Dev Disabil. 2017;71: 11–17. doi:10.1016/j.ridd.2017.09.017

57. Occelli V, Esposito G, Venuti P, Arduino GM, Zampini M. The Takete—Maluma Phenomenon in Autism Spectrum Disorders. Perception. 2013;42: 233–241. doi:10.1068/p7357

58. Fort M, Weiß A, Martin A, Peperkamp S. Looking for the bouba-kiki effect in prelexical infants. The 12th International Conference on Auditory-Visual Speech Processing. INRIA; 2013. pp. 71–76. Available: https://www.research.ed.ac.uk/en/publications/looking-for-the-bouba-kiki-effect-in-prelexical-infants-2
