# Supplementary material for: The maluma/takete effect is late: No longitudinal evidence for shape sound symbolism in the first year
Source: PLoS One. 2023 Nov 9;18(11):e0287831. doi: 10.1371/journal.pone.0287831 (PMC10635456; doi:10.1371/journal.pone.0287831)
Supplement: Attachment — Submitted filename: Review_PLOS.docx [file pone.0287831.s002.docx]

This article describes a series of studies bouba/kiki eye-gaze studies designed to document developmental changes in children between the ages of 4 and 12 months. The studies are well designed and adequately powered to detect both main effects of sound symbolism, and changes in the strength or direction of such an effect over time. The stimuli were validated as suitable bouba/kiki type sound symbolic matching in a sample of adults. A separate test was conducted to establish gaze properties of the stimuli in the age range of interest. In two critical hypothesis tests, children did not show any bouba/kiki effect (i.e., different gaze for congruent versus incongruent pairings of visual and audio stimuli), and this pattern did not differ with age, nor with additional exposure to objects and their labels. These findings differ from previously published reports of gaze behavior consistent with bouba/kiki effects within this age range, making this report an important contribution to the field. The authors discuss possible reasons for the lack of clear bouba/kiki responding, including highlighting the possibility that sound symbolic matching may take some time to emerge alongside developing language skills.

Given the extremely small number of published bouba/kiki effects in developmental samples, this report is important, timely, and adds considerably to our knowledge of how, whether and when young children might show evidence of sound-symbolic congruence. Indeed, many researchers in this field have heard myriad whispers of ‘failed’ bouba/kiki eye-gaze tests that have never been reported, suggesting a substantial ‘file drawer’ of unpublished results. Hence, despite the data aligning with the null hypothesis (i.e., no evidence of effect), the lack of evidence is informative – particularly as the study includes a series of tests. The report is clearly written, and the data are archives in an open access repository. I recommend publication with minor corrections only to enhance the clarity and completeness of the report and repository.

**Theory/Lit review/Discussion**

Page: 10

The lit review suggests the bouba/kiki effect occurs for 90% of participants in all language contexts, even though one of the cited papers in this section (Styles & Gawne 2017) proposes that the 'canonical' word forms do not work in all language contexts. This means that it may be shaped by language exposure across development. The authors do return to this point in the discussion, but there might be a way to foreshadow this point in the introduction. Consistent with this view: work by (Shang & Styles, 2017, 2023) shows that the effect can take a different form in different languages, and Woon (2018) showed that children in early primary school show a weaker bouba/kiki effect than adults in a large developmental sample.

Styles, S. J., & Gawne, L. (2017). When does maluma/takete fail? Two key failures and a meta-analysis suggest that phonology and phonotactics matter. *i-Perception, 8*(4), 1-17. doi:10.1177/2041669517724807

Shang, N., & Styles, S. J. (2017). Is a High Tone Pointy? Speakers of Different Languages Match Mandarin Chinese Tones to Visual Shapes Differently. Frontiers in Psychology, 8, 2139. https://doi.org/10.3389/fpsyg.2017.02139

Shang, N., & Styles, S. J. (2023). Implicit Association Test (IAT) Studies Investigating Pitch‐Shape Audiovisual Cross‐modal Associations Across Language Groups. Cognitive Science, 47(1). <https://doi.org/10.1111/cogs.13221>

Woon, F. T. (2018). *Linguistic sound symbolism and reading development: Sound-shape matching and predicters of reading in multilingual Singapore.* (Masters), Nanyang Technological University. <https://hdl.handle.net/10356/73469>

Page: 11

I think Ramachandran was the first to make the proposal about congruence of the movement of articulators in the bouba/kiki effect (although phrased rather vaguely). He was certainly very clear about the round shape of the lips though, so a reference to lip shape should either include his contribution, or a clear statement that more recent papers are summaries/overviews.

Ramachandran, V. S., & Hubbard, E. M. (2001). Synaesthesia—A Window Into Perception, Thought and Language. Journal of Consciousness Studies, 8(123-34).

Ohala was the first to describe the relationships between the shapes of objects and the sounds they make in the natural world. These relationships are noted in the discussion without reference to Ohala.

Ohala, J. J. (1994). The frequency code underlies the sound-symbolic use of voice pitch. In L. Hinton, J. Nichols, & J. J. Ohala (Eds.), Sound Symbolism (pp. 325-347). Cambridge: Cambridge University Press.

In general, the discussion of the paper could make clearer the different possibilities for why their data do not align with the previously published bouba/kiki effects in children under 12m. If the current study is a ***false negative***, the bouba/kiki effect may be true of the general population, but the failure to detect the effect here may be simply unlucky, OR it may reflect differences in implementation of the task (differences in stimuli, differences in test paradigm, differences in some characteristic of the population). However, the other possibility is that the current study reports a true negative, and previous results may represent ***false positives*** – there might be a robust, replicable bouba/kiki effect that is generally true of the population of children under 1year. If this is the case we would expect there to be other (perhaps unpublished) studies which also failed to observe a significant effect in early developmental samples. This possibility is hinted at in the discussion, as developmental mechanisms are suggested which might account for the gradual emergence of the effect, but the point could be made even more clearly. Styles & Gawne (2017), for example, highlight a possible ‘file drawer’ of failed studies, and encourage other authors to “bring out their dead” (p.12). If the authors believe that other explanations are more likely (e.g., individual differences in babbling across samples), the discussion could propose what particular programme of research would provide confirmatory or discriminatory evidence. This kind of clarification would help to strengthen the theoretical contribution of the paper, and provide a concrete pathway to future research on the topic.

At the moment the discussion and closing statements of the article focus instead on the (somewhat underpowered) correlation between individual babbling and possible emergence of sound symbolic looking behavior. While this is a tantalizing possibility, it would require future research to clarify whether this pattern is replicable in a well powered sample. The authors could be a bit more specific about precisely what kind of research would be needed to confirm this kind of individual difference.

**Methods**

Not sure what transliteration is being used by the team to describe the pronunciations. In the International Phonetic Alphabet the /o/ in /bobo/ is the monopthong vowel in 'got' NOT the

dipthong vowel in 'go'. I think this might be the wrong symbol if the 'written label is 'boeboe'. If the word rhymes with 'logo' and 'oboe' then the symbol should be something like /ou/

or /oʊ/. Similar problem for the other vowels I think, but hard to be sure. This is a really common problem in bouba/kiki type research, and it makes replication and metaanalysis really

hard. Please check your symbols align with the International Phonetic Alphabet, and/or to describe the stimulus words using unambiguous rhymes (e.g., 'oboe'). Where can a reader hear the

audio tokens used in the study? I couldn't find the audio recordings in the OSF repository to check.

Did the parents ever hear a member of the research team say the words aloud before the toys were sent home, or did they first encounter them written down? One brief statement suggests a parent's pronunciation was checked to for alignment to the expected wordform, but this isn’t described clearly – the procedure for the check could be added to the OSF.

**Reporting of results**

• Percentages reported without error-variance/confidence interval. Please add an index of confidence around point estimates or provide the raw frequency data in the table so the confidence can be estimated without referring to the data in supplements.

• Please include effects sizes for all effects and interactions – even when non-significant, as this is more consistent with full reporting. In some places, non-significant p-values are summarized (e.g., all p>.33). I believe most of these are reported in full in tables, but please check, as I could not find full reporting of supplementary analyses.

•Please report supplementary analyses in full. Results of key models are tabulated in text, along with mention of supplementary analyses in the text/ footnotes. If I understood correctly, the additional analyses are not reported/tabulated in full anywhere. The full analysis script is provided along with the data for analysis, but this requires a reader to run the analysis script to view the full statistical report. As the current software may become obsolete in the future, please add a supplementary analysis document to the OSF repository where the full statistical outputs can be viewed.

**Statistical Models**

Please clarify - is the 'simple' regression after removing random participant intercept still a repeated measures design? This is important because one person's responses may be correlated more closely to their own responses than to others' responses - even without the random effect being significant (i.e., people respond moderately differently to one another, but not significantly). By keeping the random factor in your model, you may actually improve the power to detect an effect. Perhaps there is different reasoning behind this decision, so I'd be happy to understand more about it - may be in a footnote or supplementary materials.

**Graphing**

Best practice in data visualization recommends that every figure summarizing the results of more than one participant include the participant N on the plot (e.g., in the legend) or in the

Figure description, so that a reader doesn't have to go searching for the info in other parts of the article. Please add N to each graph or each age group. This also helps a reader to get an overview of whether the graph includes incomplete data across longitudinal samples (e.g., more samples at earlier than timepoints) or includes only those samples who were tested across all timepoint (i.e., no dropouts included in graph).

Best practice in data viz is to include individual data points where possible, as well as an indication of confidence estimates about any point estimate drawn from more than one data point. Figure 3 uses bars to represent percentage of responses. This obfuscates the precise computation from 8 trials down to four bars: are we seeing the average value across the group for the individuals’ percentages in each condition (i.e., only possible values for each person are 100%, 50%, 0%)? Or something else? The authors could consider an alternative way of graphing to show individual binary responses – for example, dot plots - this would help to visually communicate the structure of the data, and make clearer that participants responses differ substantially across different items (e.g., almost at chance for ‘cheechee’)

**Archive of Data, Code and Analysis**

**Code for running the studies** not yet provided in OSF. Please provide the scripts for the adult study (EPrime) and the eye-gaze studies (Habit). Including these would help make the study (or future adaptations) more replicable in the future.

**3D wireframes.** Since the test objects were 3d printed, it would be great to include the wireframes used for 3D printing of the objects, or a link to a digital file on a maker-site. This would enhance future replicability of the project.

**Visual Stimuli** For future replicability, please add photographs of the 3D shapes as used on screen in the study.

**Audio Stimuli.** One of the challenges of bouba/kiki studies internationally is knowing precisely what participants heard. One solution to this problem is accurate transcription in IPA, but as became clear in the metaanalysis by Styles & Gawne (2018), the notations provided in papers do not always align with strict IPA transcription. To allow greater transparency, please document the audio files in the OSF. As audio recording quality can differ substantially, including the audio files would readers to know what participants actually heard, including whether the content of the audio files can be easily heard by study participants. Archiving would also allow potential replicability into the future.

**Data** **files** in archive have several different structures. It would be super helpful to include a code-book or a wiki explaining the different data files, their origin (what software & version?), and what each column represents in each file. Some of the Habit files share a structure, but for a casual viewer, it is important to know which columns are user-defined (e.g., Family and ID), and which of the automated columns are used in analysis. The Individual differences data file is particularly rich, but it is not clear where to look for the coding scheme if you start by looking at the data (the survey form I think?).

**Surveys**

Current archive only includes the survey from T1, but the text mentions that later surveys have different questions about babbling. Please include the full set of surveys for the different timepoints, with the precise questions answered by parents, and any protocol for implementation if additional questions were asked by a researcher.

• The archive appears to only include data from T3 – which appears in the report – but as data were collected at T1 and T2, it would be better to include a complete record of survey data at all three timepoints. Alternatively, if those data are reserved for another project, this should be described along with a link to where the data are archived separately. OSF allows archives to be created before being made public so if an embargo currently exists, a link can be provided for future accessibility.

• Many of the questions in the survey are not reported in the current writeup. There can be many reasons for not reporting all outcome measures, including that the bouba/kiki study reported here was part of a larger research project, which will be reported in full elsewhere. It is important to disclose the purpose for collecting tis data so a reader can know whether there was more exploratory analysis than the results reported here (i.e., multiple possible outcome measures were collected and only significant ones are reported in the current paper) or pre-conceived hypothesis tests. If exploratory, the analysis should be reported as such, along with the results of all non-significant tests, so that the probability values can be understood in context. If the study was preregistered, please clarify this in the main text of the study description so that the prereg can be readily identified.

**Minor points (these are all annotated as comments on the PDF):**

Page: 3

Ethics approval number not yet given

Page: 12

Also relevant: the Maurer procedure was non-blind to the experimenter, so children might be picking up other subtle paralinguistic or prosodic cues when identifying the expected response.

Page: 15

Were demographics not collected at the time of test? or not retained?

Page: 18

Please add a measure of confidence around this point estimate (e.g., CI), or clarify

where can we find details of the analysis and how it was implemented?

Page: 19

This graph would be better if it were possible to view individual data points (e.g., a geompoint layer overlapping with/alongside the bar), or distributions in the format of density plots

Page: 20

Table would be more informative if it included an indication of error/variance about the % listed - e.g., 95% CIs

Page: 21

Which written form? /bobo/ or "boeboe"?

More detailed description needed - same as the first analysis (with random factor for participants?) or the second analysis?

More detail needed (e.g., in supplement or code repository)

NB. Shang and Styles (2017) uses 3D shapes, as do Fryer, Freeman and Pring (2014) – albeit with touch alone.

Fryer, L., Freeman, J., & Pring, L. (2014). Touching words is not enough: how visual experience influences haptic-auditory associations in the "Bouba-Kiki" effect. *Cognition, 132*(2), 164-173. doi:10.1016/j.cognition.2014.03.015

Page: 22

Great level of detail in demographics!

Great use of Open Archiving :)

Page: 23

Not clear whether this developmental survey was intended for exploratory analysis of the results of the current study, or simply to characterize the kids in the study according to their general development. Alternatively, these data might have been collected for use in a different/larger study. Please clarify.

It looks like only one version of the survey is currently in the archive - it would be great to see the full set there :)

Page: 24

Are the demographics and language questionnaires and the resulting data archived?

What is the degree of visual angle subtended by the two stimuli? this is important to understand, as it may mean that children need to make just a saccade or a head movement to switch from one image to the other. This becomes important when considering the duration of looks included/discarded.

Please archive the Habit code

Page: 25

Are the results from the CDI archived? (full data best, but score for each child also valuable if full data is embargoed for another study)

Page: 26

Author: ssspsymac10Subject: Text Box Date: 11/1/23, 1:53:41 PM

I'd like to hear more about your reasoning for the exclusion threshold, and whether this was an a priori decision made data-blind (following an established protocol from previous study, before seeing current data), a condition-blind decision (applied after screening the data in a format where the condition of each trial was blind to the researcher making the decision), a results blind decision

(made after a screening process where the condition of each trial was known, but before any between condition statistical comparison had been conducted), or a post-hoc decision after a

preliminary analysis was conducted.

Author: ssspsymac10Subject: Highlight Date: 11/1/23, 1:45:48 PM

Author: ssspsymac10Subject: Text Box Date: 11/1/23, 2:07:59 PM

Perhaps I am missing something about the structure of the model, but without inclusion of a random factor for individual, this treats every visit as though it is a different group of

children, and there is no repeated measures structure in the analysis - am I right? RM analyses usually have more power to detect within subjects effects.

Please refer your reader to Table for full statistical report

Confusing wording here - consider rephrasing to "no significant main-effect of nonword type was observed."

Page: 27

Please provide full statistical report in text, table or supplement

Table 2 should be mentioned in the text so a reader knows where to look for the full statistical output

Please add effect size for all effects including n.s. ones as this allows for future metaanalyses

Page: 28

Please include sample size on figure, in Title or Note

Page: 29

Suggested rephrasing: For a detailed introduction to this approach we refer the reader to ...

Please unpack this a little for the reader - what does this mean for interpretation of the null hypothesis?

Page: 30

Please add a supplement or report in text/a table

Consider rephrasing to “no significant effect was found” as it is a bit weird to say an effect (which was non significant) was found, rather than a failure to detect a significant effect. This is a minor matter of stylistics, but is a little clearer for a reader.

Please include all analyses - including non-significant ones - in the archive for the article or supplementary materials.

Page: 31

How were decisions made about which variables to explore, and at what timepoints? Was this a pre-planned analysis before data were seen? or exploratory (both are ok but good to specify so that they can be interpreted appropriately

at what timepoint? this is not included at timepoint 1, and I wasn't able to find a version including these questions in the archive

entered into the model separately? or combined into a single score of some kind?

First mention of this in this section - also derived from factor analyses, or a raw score from original survey?

Full statistical reports for the factor analyses not yet in the Archive? Please add a supplement

Page: 32

unclear reasoning here... I understand that there were different surveys at different time points, but am I correct that only visit 3 is included in this section of the paper? Both for the survey and for the test data? It would be great to hear more about the reasoning for only testing T3.

Important to note in an exploratory analysis of this kind :)

not significant if the alpha was .05. Best to avoid the expression 'marginal' if you are doing null hypothesis statistical testing as it makes statisticians really mad.

Perhaps I wasn't following clearly, but I wasn't sure how time was implemented for each of these correlations. The surveys were conducted at 3 time points, as were the gaze direction tests.

Were the factor analyses run separately for each time point and then correlations were tested only for synchronous correspondence between survey and test? Or wwere all score across time

points collapsed into a single measure of individual difference?

Page: 33

The data collected at all 3 timepoints is interesting to other researchers in the field so unless reserved for another study, it would be better to report values for all survey data at all

time points if possible.

This text is unclear. Perhaps better to split into two tables, or two columns in one table. One for table/column could be for binary milestones, where the number reported is % of children,

and one table/column where the number is the mean score.

Please present these data together with some indication of error/variance e.g., Confidence Intervals

Page: 34

Where are the mean scores for the EFA reported/Archived?

Page: 36

Please unpack this a bit more - was this a mistake by the parent? by the experimenter? or something else?

Was this based on an a priori power analysis or some other rubric?

Page: 38

This analysis seems different from the previous where the analysis was conducted in terms of shape first and label second. Did I miss something?

Need some data display to contextualize the results relative to the previous study (even if non significant

Please add full report in supplement/OSF

Page: 39

effect size?

Page: 40

A little bit misleading given the lack of statistically significant difference

Page: 41

See also the dissertation by Woon 2018 where 9-year olds show less tendency for sound symbolic responding than adults.

Page: 42

rephrase?

See also Shang & Styles (2018, 2023)for differences in the nature of sound symbolism for langs with different acoustic features.

Page: 43

May be better to rephrase with optimistic framing - e.g., although it would not survive correction for multiple comparisons in the current study, it suggests a new target for hypothesis testing in future work.

But see Bottini, Barilari & Collignon (2017), Shang & Styles (2017) for strong effects in 3D (albeit different study designs)

It is also worth noting that there may be a large 'file drawer of unreported 'failed' studies within this age range, thereby masking whether the effect is robust and reliable at this early

age.
